# Identification of Inhibitors against *Mycobacterium tuberculosis* Thiamin Phosphate Synthase, an Important Target for the Development of Anti-TB Drugs

**DOI:** 10.1371/journal.pone.0022441

**Published:** 2011-07-26

**Authors:** Garima Khare, Ritika Kar, Anil K. Tyagi

**Affiliations:** Department of Biochemistry, University of Delhi, New Delhi, India; Universita di Sassari, Italy

## Abstract

Tuberculosis (TB) continues to pose a serious challenge to human health afflicting a large number of people throughout the world. In spite of the availability of drugs for the treatment of TB, the non-compliance to 6–9 months long chemotherapeutic regimens often results in the emergence of multidrug resistant strains of *Mycobacterium tuberculosis* adding to the precariousness of the situation. This has necessitated the development of more effective drugs. Thiamin biosynthesis, an important metabolic pathway of *M.tuberculosis*, is shown to be essential for the intracellular growth of this pathogen and hence, it is believed that inhibition of this pathway would severely affect the growth of *M.tuberculosis*. In this study, a comparative homology model of *M.tuberculosis* thiamin phosphate synthase (MtTPS) was generated and employed for virtual screening of NCI diversity set II to select potential inhibitors. The best 39 compounds based on the docking results were evaluated for their potential to inhibit the MtTPS activity. Seven compounds inhibited MtTPS activity with IC_50_ values ranging from 20 – 100 µg/ml and two of these exhibited weak inhibition of *M.tuberculosis* growth with MIC_99_ values being 125 µg/ml and 162.5 µg/ml while one compound was identified as a very potent inhibitor of *M.tuberculosis* growth with an MIC_99_ value of 6 µg/ml. This study establishes MtTPS as a novel drug target against *M.tuberculosis* leading to the identification of new lead molecules for the development of antitubercular drugs. Further optimization of these lead compounds could result in more potent therapeutic molecules against Tuberculosis.

## Introduction

Thiamin pyrophosphate (TPP), an important cofactor for several enzymes such as pyruvate dehydrogenase, transketolase, 2-oxoglutarate dehydrogenase and acetohydroxyacid synthase, is involved in cleaving the carbon-carbon bonds adjacent to a carbonyl group thus playing an important role in various processes such as glycolysis, TCA cycle, pentose phosphate pathway and metabolism of branched chain amino acids [1]–[5]. Hence, maintaining the adequate levels of TPP and TPP-utilizing enzymes is essential to all known cellular life forms. However, while most microorganisms can synthesize thiamin de novo, animals and many fungi require thiamin or its immediate precursors as a supplement in their diet. Thiamin phosphate synthase (TPS) is a bacterial protein involved in the biosynthesis of thiamin pyrophosphate (TPP), the active form of thiamin (vitamin B1) [6]. Thiamin phosphate synthase (ThiE) of *Mycobacterium tuberculosis*, the product of the last gene of thiamin biosynthetic pathway (*rv0414c*), has been identified as an *in vivo* essential enzyme for the pathogen, emphasizing its importance as a potential drug target [7]. Unlike most microorganisms, *M.tuberculosis* does not contain the genes for thiamin salvage pathway and transporters [8] further substantiating the importance of thiamin biosynthesis for the pathogen's survival thus making TPS an attractive target for the development of antitubercular drugs. It catalyzes the substitution of pyrophosphate of 2-methyl-4-amino-5-hydroxymethylpyrimidine pyrophosphate (HMP-PP) by 4-methyl-5-(beta-hydroxyethyl) thiazole phosphate (Thz-P) to yield thiamin phosphate in the thiamin biosynthesis pathway, which is further phosphorylated to thiamin pyrophosphate (TPP) [6], [9], [10]. Although, TPS represents a very important enzyme for the survival of microorganisms, till date it has not been exploited as a drug target and no inhibitor against it has been identified. In this work, we generated a three dimensional homology model for *M.tuberculosis* thiamin phosphate synthase (MtTPS) and carried out virtual screening with National Cancer Institute (NCI) diversity set II containing 1541 compounds with nonredundant pharmacophore profiles against the substrate cavity of the enzyme. Further the shortlisted compounds were evaluated for the inhibition of thiamin phosphate synthase activity in vitro as well as the growth of *M.tuberculosis* in broth culture. This study for the first time emphasizes the importance of MtTPS as a target for the development of new interventions against *M.tuberculosis.*

## Results

### Cloning, Expression and Purification of MtTPS

*thiE (rv0414c)* was cloned in pET28a and expression was carried out as described in materials and methods. The localization of the expressed protein was analyzed by SDS electrophoresis using a 12.5% polyacrylamide gel. A distinct band of MtTPS was observed at an apparent molecular weight of ∼29 kDa with the entire recombinant protein localizing in the insoluble fraction, when the induction was carried out at 37°C (data not shown). In order to enhance the solubility of MtTPS, its expression was also evaluated at 25°C and 15°C. The induction at 25°C also resulted in the localization of majority of the protein in the insoluble fraction (data not shown), however, at 15°C, 10% of the expressed MtTPS localized in the soluble fraction (Figure S1a, lane 7), which was purified by strep - tactin affinity chromatography as described in materials and methods and the purified enzyme was found to be ∼80% pure (Figure S1b, lanes 5–10).

### Determination of the enzymatic activity of MtTPS

*E.coli* ThiD or HMP-kinase was purified to ∼95% purity (Figure S2) and was used for the enzymatic synthesis of HMP-PP that is required as one of the substrates for TPS. The activity of MtTPS was measured by the thiochrome assay as described in materials and methods. The specific activity of MtTPS was determined to be 1.82 nmoles/min/mg as opposed to 65 nmoles/min/mg reported for *E.coli* TPS (EcTPS) [11], [12]. The Michaelis Menten constants or the K_m_ values of the substrates HMP-PP and Thiazole-P were determined to be 5 µM and 14 µM, respectively (Figure S3).

### Homology modeling of MtTPS

The preliminary similarity search for MtTPS amino acid sequence by using NCBI BLAST server [13] against the PDB database exhibited the highest homology with thiamin phosphate synthase from *Pyrococcus furiosus* (PfTPS, 1XI3 – unpublished data) and *Bacillus subtilis* (BsTPS, 2TPS) [14], [15] (data not shown). Multiple sequence alignment by using ClustalW [16] displayed 34.3% identity and 67.6% similarity between MtTPS and PfTPS, while MtTPS and BsTPS shared 28.8% identity and 66.6% similarity (Figure S4). Based on the BLAST results, the top four hits (PfTPS: PDB ID - 1XI3 and BsTPS: PDB ID - 2TPS, 1G4P and 1G4E) were analyzed. The structures with PDB ID - 2TPS and 1G4P were not considered further for model building since 2TPS represented the crystal structure of BsTPS complexed with the reaction products thiamin phosphate and pyrophosphate [14], while 1G4P represented the crystal structure of an S130A mutant of BsTPS complexed with 4-amino-2-trifluoromethyl-5-hydroxymethylpyrimidine pyrophosphate (CF_3_HMP-PP) [15]. 1XI3 and 1G4E represented structures without any ligand and therefore were considered suitable for generating the homology model for MtTPS. Based on the crystal structures of PfTPS and BsTPS, three-dimensional homology models for MtTPS were generated by two online protein modeling servers: SWISS-MODEL [17], [18] and ESyPred3D [19]. Table 1 shows the total energy calculated for the top models generated by these softwares as analyzed by using DeepView-Swiss pdb Viewer [20]. The MtTPS model generated by using SWISS-MODEL server [17], [18] with PfTPS structure as the template was selected for further work due to the highest overall negative energy of this structure (TPS_1X13). Besides, MtTPS exhibited higher sequence similarity with PfTPS as compared to BsTPS. Further, the stereochemical quality of the model was assessed by PROCHECK [21]. The results showed that the backbone Φ and Ψ dihedral angles of 87.5%, 8.7%, 2.2% of the residues were located in the most favoured, additionally allowed and generously allowed regions of the Ramachandran plot, respectively, with only 3 residues (1.6%) in the disallowed regions (Table 2, Figure S5). It was verified that none of the residues belonging to the disallowed region were located in the active site, thus validating the overall stereochemical quality of the MtTPS model. Further, the model was evaluated by using PROSA [22], [23], PRO-Q [24], VERIFY3D [25], [26], and ERRAT [27]. The results obtained from these programs imply that the residues in the model are placed in very good overall configuration (Table 2 and Figure S6, S7). The MtTPS model was subjected to energy minimization by using the AMBER9 software [28] by applying the forcefield ‘ff03’ and 500 cycles of minimization.

**Table 1:** Energy calculations for top models of MtTPS generated by SWISS-MODEL and ESyPred3D.

	Homology model by using 1XI3 as template (TPS_1XI3)	Homology model by using 1G4E as template (TPS_1G4E)
**SWISS-MODEL structure**	−5577.096	−4400.27
**ESyPred3D structure**	596.321	850.032

**Table 2:** Evaluation of MtTPS homology model by using PROCHECK, PROSA, PRO-Q, VERIFY3D and ERRAT online protein structure evaluation tools.

*PROCHECK (*R.C.Plot analysis)	TPS_1XI3
**1. Favoured**	87.5%
**2. Additionally allowed**	8.7%
**3. Generously allowed**	2.2%
**4. Disallowed**	1.6% (3 residues)
***PROSA***	
**1. Z Score**	−7.08
**2. Local model quality**	No positive regions
***PRO-Q***	
**1. LG score**	4.551
**(>1.5 fairly good, >2.5 very good, >4.0 extremely good)**	(extremely good)
**2. MAX-SUB score**	0.460
**(>0.1 fairly good, >0.5 very good, >0.8 extremely good)**	(close to very good)
***VERIFY 3D***	
**Number of residues with score >0.2**	90.45% (pass)
***ERRAT***	
**Overall quality factor**	61.611%

### Salient features of MtTPS structure

**Figure 1:**
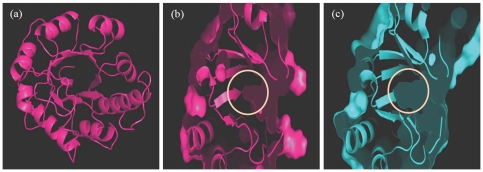
Comparative homology modeling based three-dimensional structure of MtTPS. (a) The overall structure of MtTPS showing the characteristic triosephosphate isomerase fold. The binding site groove of MtTPS (b) and PfTPS (c) is shown and the depth of the binding site groove is encircled. The structure is shown from a side view rotated 90 degrees along the vertical axis with respect to the top view. The figures were prepared by using Pymol Molecular viewer [56].

Thiamin phosphate synthase belongs to the family of α/β proteins with a triosephosphate isomerase fold (TIM barrel) having an inner core of 8-α helices and eight parallel β-strands tilted approximately 45° from the axis of the barrel (Figure 1a). The BsTPS active site is primarily formed by the three loops spanning the central cavity of the structure and is covered by the largest loop [14], [15]. The substrate binding groove of the TPS lies in the area surrounded by the β-barrel structure present in the central area of the TIM-fold structure of TPS. The residues from the β-strands pointing towards the interior of the barrel form a hydrophobic core and provide interactions for binding to the substrate [14], [15]. Upon visual comparison of the active site groove, it was found that it is narrower and less deep in the structure of MtTPS as compared to the crystal structures of PfTPS and BsTPS (Figure 1b and 1c, substrate binding groove of BsTPS not shown).

### Virtual screening for the identification of inhibitors against MtTPS

**Figure 2:**
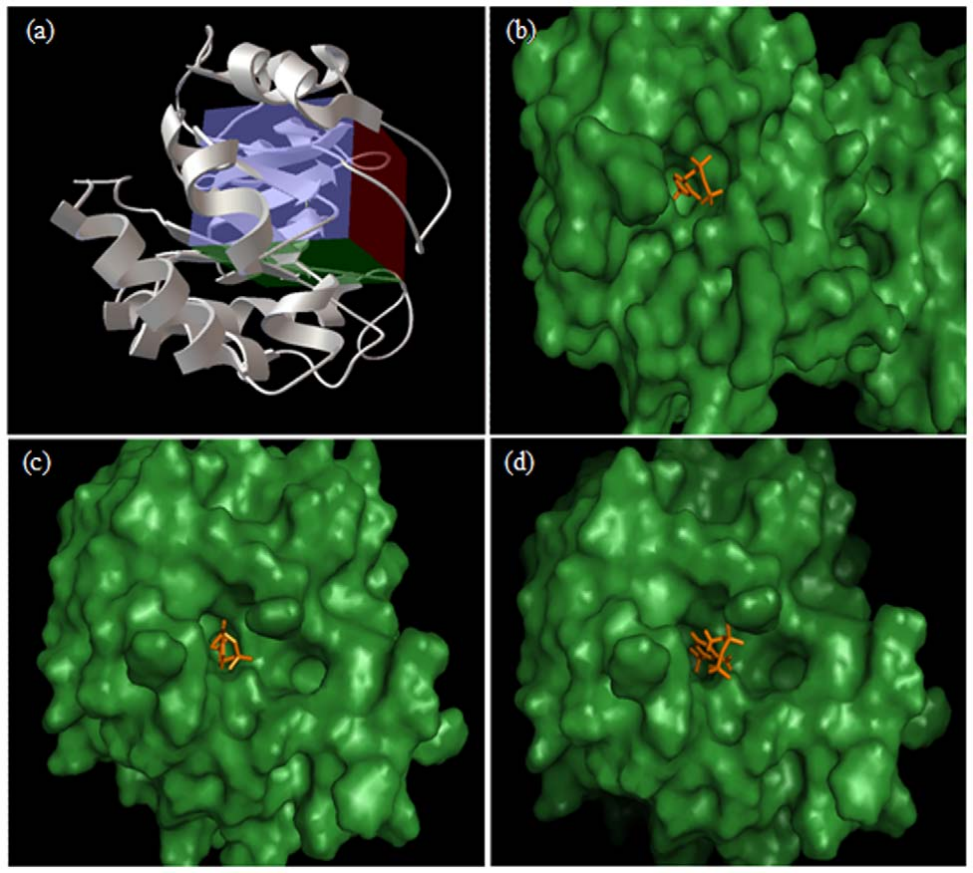
Verification of the docking procedures of Autodock4 and DOCK6. (a) A Ribbon representation of MtTPS structure is shown in a side view rotated 90 degrees along the vertical axis with respect to the top view. The grid used for the docking of ligands is shown in a box representation. (b) The ligand CF_3_HMP-PP bound at the active site of BsTPS in the X-ray crystal structure (PDB ID- 1G4P). Ligand docked by Autodock4 (c) and DOCK6 (d) at the active site of MtTPS. The ligand is shown in orange stick model. Figure (a) was prepared by software Autodock4 while the rest of the figures were prepared by using Pymol Molecular viewer [56].

NCI diversity set II containing 1541 diverse compounds was virtually screened against the MtTPS model with the substrate binding groove or the HMP-PP binding site as the target for docking by using Autodock4 [29] and DOCK6 [30] (Figure 2a). This library set was selected owing to the tremendously diverse nature and pharmacologically desirable features of the compounds in this collection. To assess the accuracy of the docking procedures and the parameters employed in the docking, CF_3_HMP-PP (an analog of HMP-PP, the natural substrate of TPS) was docked to the MtTPS structure and was compared with the X-ray structure of BsTPS bound to this ligand (Figure 2b). The result verified the accuracy of the docking procedures and the parameters employed as seen by the correct docking as well as the orientation of the ligand docked by both the softwares (Figure 2c and 2d). Scoring system used by Autodock4 [29] and DOCK6 [30] are based on different algorithms and a direct comparison of these results is not appropriate. Hence, to provide a comparable platform to enable a direct comparison of the results generated by the two docking softwares, X-Score [31] was used. X-Score [31] takes the output files of the docking softwares as input file for recalculation of the docked conformation of the molecules and re-ranks the molecules according to its own algorithm. The best 40 compounds based on the docking results and re-ranking were requested from National Cancer Institute - Developmental Therapeutics Program (NCI-DTP), out of which 39 compounds were obtained and experimentally screened in order to assess their ability to inhibit the MtTPS activity. Table S1 shows the list and ranking of the compounds obtained from NCI-DTP, while the chemical structures of the compounds are given in supporting information S1. It may be stated that NCI diversity set II is comprised of the molecules having pharmacologically desirable features. It was further verified by the fact that during X-Score analysis, when the ligand pre-screening option was enabled (to assess drug-like properties), none of the molecules in this set was excluded from the scoring (data not shown).

### Evaluation of inhibitory potential of the compounds against MtTPS activity

**Figure 3:**
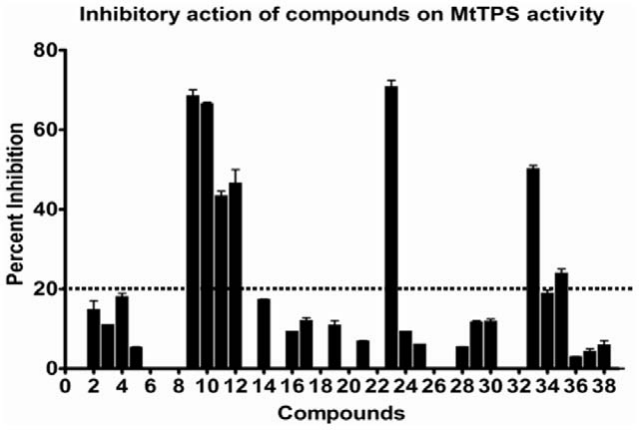
Evaluation of the compounds for their potential to inhibit the activity of MtTPS. Bar diagram represents percent inhibition of MtTPS activity in the presence of compounds at a concentration of 100 µg/ml. The data depicts the values as mean ± S.E. of two separate experiments carried out in duplicates.

The potential of the compounds to inhibit MtTPS activity was measured at a concentration of 100 µg/ml in the normal assay conditions as described in the materials and methods section. Among the 39 compounds evaluated, 25 exhibited inhibition of the MtTPS activity with seven compounds (9, 10, 11, 12, 23, 33 and 35) exhibiting greater than 20% inhibition and were therefore selected for further studies (Figure 3). IC_50_ values of the selected seven compounds were determined at the HMP-PP concentration of 5 µM (the K_m_ value) by measuring the inhibition of MtTPS activity in the presence of varying concentrations of these compounds (Table 3). It was observed that compounds 23 and 9 exhibited the highest inhibition of MtTPS activity with IC_50_ values of 20 µg/ml and 34 µg/ml, respectively, whereas the IC_50_ value of compound 10 was determined to be 70 µg/ml. Compounds 12 and 33 displayed 50% inhibition of the activity at a concentration of 100 µg/ml, while the IC_50_ values of compounds 11 and 35 were estimated to be >100 µg/ml.

**Table 3:** IC_50_ and MIC_99_ values of the top seven compounds.

S. No.	Compd.	NSC number	IC_50_ (µ/ml)	MIC_99_ for *M.smegmatis* (µg/ml)	MIC_99_ for *M.tuberculosis* (µg/ml)
1	9	33472	34	32% inhibition at 750 µg/ml	6
2	10	37168	70	No inhibition	750
3	11	42199	>100	125	250
4	12	50648	100	45% inhibition at 750 µg/ml	32% inhibition at 750 µg/ml
5	23	116720	20	500	300
6	33	327702	100	150	162.5
7	35	338963	>100	No inhibition	125

### Determination of MIC_99_ values of selected compounds against *M.smegmatis* and *M.tuberculosis*

**Figure 4:**
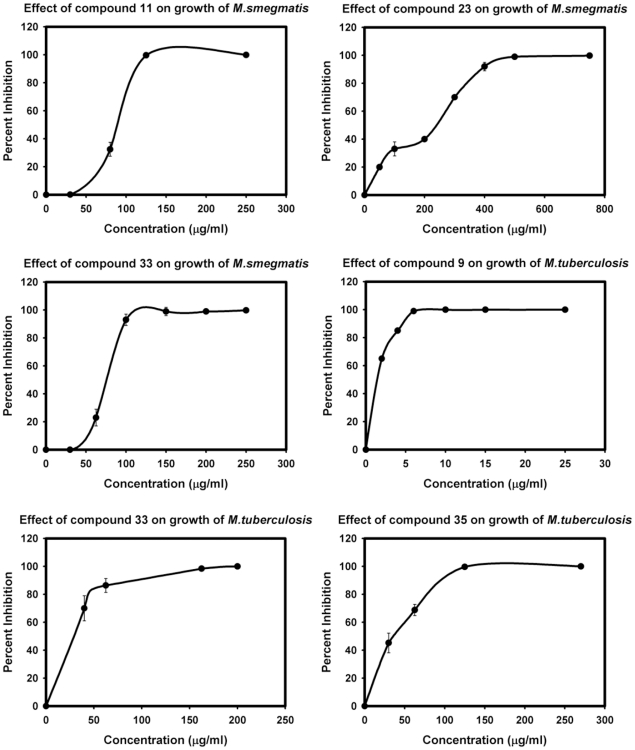
Effect of compounds on the viability of *M.smegmatis* and *M.tuberculosis*. The data depicts the values as mean ± S.E. of two separate experiments.

To quickly obtain the preliminary information about their inhibitory potential, the seven active compounds were first examined for their inhibitory activity against *M.smegmatis*, a fast growing species of mycobacteria by using broth macrodilution method and the results are summarized in table 3. Compounds 10 and 35 exhibited no inhibition of *M.smegmatis* growth even upto a concentration of 750 µg/ml. Compounds 9 and 12 exhibited 32% and 45% inhibition of *M.smegmatis* growth, respectively, at a concentration of 750 µg/ml. Compounds 11 and 33 showed the highest inhibition of *M.smegmatis* growth with MIC_99_ values being 125 µg/ml and 150 µg/ml, respectively. Compound 23 exhibited a moderate inhibition of *M.smegmatis* growth with an MIC_99_ value of 500 µg/ml (Table 3, Figure 4).

**Figure 5:**
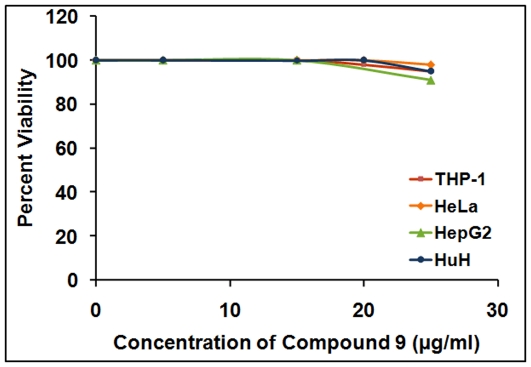
Evaluation of compound 9 for its cytotoxic effect on various cell lines. Cell viability of THP-1, HeLa, HepG2 and HuH cells in the presence of varying concentrations of compound 9.

After the preliminary studies with *M.smegmatis*, these compounds were evaluated for their potential to inhibit the growth of *M.tuberculosis*, the pathogen that causes tuberculosis. The results revealed that compound 12 showed only marginal growth inhibition of ∼32% even upto a concentration of 750 µg/ml. Compounds 11, 23 and 10 displayed MIC_99_ values of 250 µg/ml, 300 µg/ml and 750 µg/ml, respectively with compounds 35 and 33 exhibiting a significant inhibition with MIC_99_ values of 125 µg/ml and 162.5 µg/ml, respectively. However, compound 9 displayed a very significant inhibition with an MIC_99_ value of 6 µg/ml (Table 3, Figure 4). These results were verified several times to rule out any possibility of the presence of impurity or contamination in the culture, which might be inhibitory to mycobacterial growth. In addition, to rule out the action of compound 9 due to any toxic effect on the cells, we examined the cytotoxicity of compound 9 on various cell lines including THP-1, HeLa, HepG2 and HuH cells by trypan blue exclusion method. We observed that compound 9 showed no significant cytotoxic effect on any of these cells up to a concentration of 25 µg/ml (Figure 5).

## Discussion

Thiamin biosynthetic pathway represents an attractive target for the development of antitubercular drugs due to its essential requirement for the survival of *M. tuberculosis.* The *thiE* specifically figures amongst the index of genes reported to be essential for the *in vivo* growth of *M.tuberculosis* [7]. Besides, in a path breaking study by employing transposon site hybridization (TraSH), Sassetti et al. have also identified thiamin biosynthetic genes to be essential for *M.tuberculosis* in vitro [32]. Thiamin is indispensable for the activity of carbohydrates and branched chain amino acid metabolizing enzymes [5], therefore, it is an essential cofactor for all organisms. Its absence severely limits the availability of carbon source required for the growth of the organism. It has been illustrated clearly that thiamin auxotrophy, for example, in the case of *Arabidopsis*, leads to loss of viability [33], [34]. However, this is not a stand-alone example as it has also been shown in many other cases of auxotrophy. For example, inositol auxotrophs of *Neurospora* [35] and inositol – requiring species of yeast die logarithmically when starved for inositol [36], [37], [38]. Saturated fatty acid auxotrophs behave similarly during fatty acid starvation [39] and biotin or pantothenate requiring organisms when starved for their requirements do not survive either [40], [41], [42]. More close to the context of this study, NAD^+^ auxotrophy is shown to be bacteriocidal for *M.tuberculosis* [43]. Indeed, the salvage and transport pathways present in most bacteria can sometimes complement de novo biosynthesis and cripple the value of such an auxotrophic situation. However, *M. tuberculosis* represents a unique case in that it lacks both the salvage pathways as well as transporters of thiamin [8], which has provided unique importance to thiamin biosynthesis as a target for developing new drugs against this pathogen. The absence of thiamin biosynthesis genes in humans makes it even more exclusive target for the development of new TB drugs. Thiamin phosphate synthase or ThiE catalyzes the final step in the synthesis of thiamin phosphate. Recently, a number of inhibitors against various targets have been successfully identified by employing structure based drug designing using virtual screening [44], [45], [46]. Hence, the importance of this approach in discovering new lead molecules and drugs against various diseases cannot be overemphasized.

**Figure 6:**
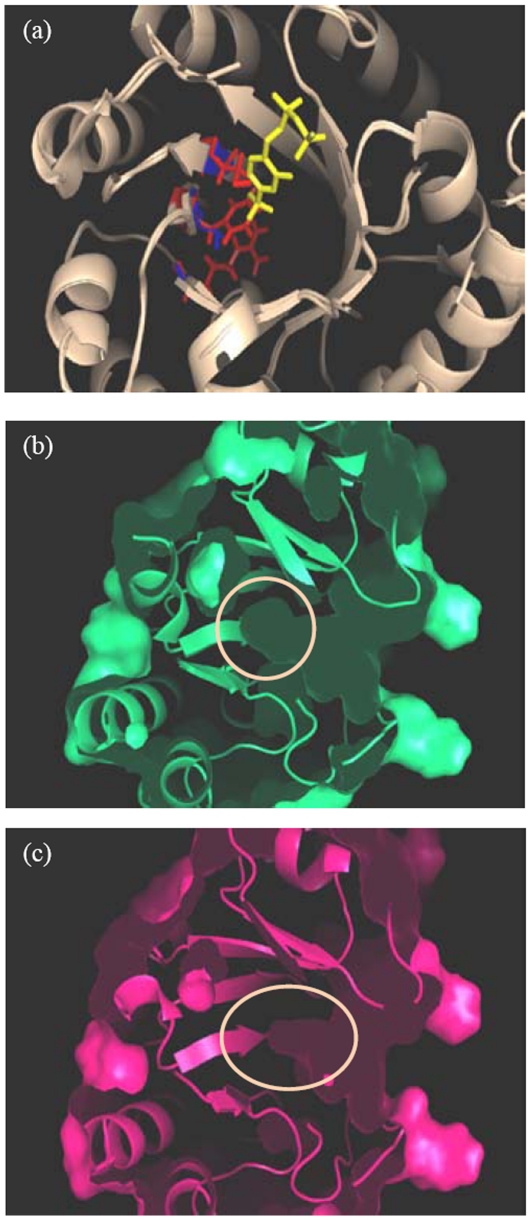
Differences in the binding site of MtTPS. (a) Comparison of the residues at the binding site groove. The structures of MtTPS and PfTPS are superimposed (wheat colour), CF_3_HMP-PP is bound at the binding site (yellow colour), residues Cys^139^, Phe^174^ and Arg^194^ of MtTPS (red) and corresponding Gly^125^ ,Val^158^, Gly^178^ of PfTPS (blue) are shown in stick model. (b) Molecular surface representation of the mutated MtTPS structure. In-silico substitutions of Cys^139^, Phe^174^ and Arg^194^ in the MtTPS structure by Gly, Val and Gly resulted in a much deeper active-site groove than in the original structure. (c) Molecular surface representation of the original MtTPS structure. The depth of the groove is encircled. The figures were prepared by using Pymol Molecular viewer [56].

In this study, a three – dimensional homology model of *M.tuberculosis* thiamin phosphate synthase was constructed by using the X-ray crystal structure of thiamin phosphate synthase from *Pyrococcus furiosus*, since the crystal structure of MtTPS was not available. Examination of the substrate binding groove of MtTPS, PfTPS and BsTPS revealed that the substrate binding groove of MtTPS is narrower and shallower as compared to its counterparts in PfTPS and BsTPS. A comparison of the residues in the binding site groove showed that although most residues in this region were identical in case of all the three enzymes, three of the residues were distinctly different in case of MtTPS. Gly^125^, Val^158^ and Gly^178^, the short chain residues present in PfTPS and the corresponding residues Gly^136^, Val^171^ and Gly^191^ in BsTPS are replaced by long chain residues namely Cys^139^, Phe^174^ and Arg^194^ in the case of MtTPS, which may possibly be responsible for the narrower binding groove of the latter (Figure 6a). This lack of depth of binding site groove in case of MtTPS may be responsible for its reduced affinity for substrate binding. Infact, when residues Cys^139^, Phe^174^ and Arg^194^ of MtTPS were in silico mutated to Gly, Val and Gly, respectively, it resulted in much deeper and broader substrate binding groove (Figure 6b and 6c). *E.coli* TPS (EcTPS) also has short chain residues in these positions at the active site as represented by Ala^127^, Val^162^ and Ser^182^. Although, the activity of PfTPS and BsTPS is not reported in the literature, EcTPS has 30-40 times higher activity than MtTPS substantiating the notion that the presence of long chain residues in case of MtTPS leads to reduction in its enzymatic activity. However, it must be mentioned that a relatively lower activity exhibited by MtTPS in comparison to its *E.coli* counterpart is well suited for the slow growing nature of *M.tuberculosis*.

**Figure 7:**
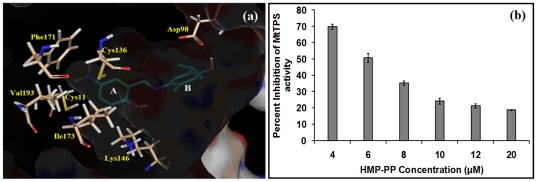
Interaction of compound 9 with the active site of MtTPS and its mode of action. (a) Binding mode of compound 9 (NSC33472) at the active site of MtTPS. The figure was prepared by using Pymol Molecular viewer [56]. (b) Inhibition of MtTPS activity by compound 9 in the presence of varying concentrations of HMP-PP.

Computational screening approach was employed to identify potential small-molecule inhibitors of MtTPS from the NCI diversity set II comprising of 1541 compounds. Out of the 39 selected compounds evaluated for their inhibitory activity, compound 9 (4-{[(2-hydroxy-5-nitrophenyl)methylidene]amino}-5-methyl-2-(propan-2-yl)phenol), 33 (3-benzylsulfanyl-phenanthro[9,10-e][1], [2], [4]triazine) and 35 (Coumarin, 7-[[4-chloro-6-(diethylamino)-s-triazin-2-yl]amino]-3-phenyl-) were identified as potential inhibitors of *M.tuberculosis* growth. All these compounds exhibited inhibition of MtTPS enzymatic activity as well as the growth of *M. tuberculosis* in broth culture. However, compound 9 exhibited the highest efficacy with an MIC_99_ value of 6 µg/ml. In addition, it did not exhibit any significant toxicity in various cell lines till a concentration of 25 µg/ml and also adhered to the Lipinsky rules for drug-likeness. The binding mode of compound 9 provided key insights into the likely binding sites (Figure 7a). The compound 9 or NSC 33472 is docked at the large hydrophobic pocket at the active site of MtTPS. The aromatic ring A is placed in a hydrophobic environment surrounded by Ile^173^, Val^193^ and Phe^171^ while the two oxygen atoms of the nitro group appear to be making hydrogen bonds with the hydrogen atoms of the adjacent Cys^136^ and Cys^11^ both present within 2.5Å distance from the oxygen atoms. Moreover, the hydroxyl group of the aromatic ring B can form hydrogen bond with the carboxyl group of Asp^98^ present at a distance of 1.78Å. Inhibition of MtTPS by compound 9 in the presence of varying concentrations of the substrate HMP-PP (Figure 7b) showed that an enhancement in the concentration of the substrate causes a decline in the inhibition and vice versa, which clearly indicates that compound 9 inhibits MtTPS by competing with HMP-PP for binding at the active site thus substantiating the docking results.

In conclusion, we have identified a promising lead molecule (compound 9) for the development of sterilizing agents against *M.tuberculosis* and further efforts are being made to optimize and enhance the inhibitory potency of this lead compound. The other two weakly active compounds (compound 33 and 35) would also be exploited further to design more potent molecules. Efforts will also be diverted towards the identification of new compounds having a better inhibitory potential. In addition, we anticipate that selective inhibition of thiamin biosynthetic enzymes would prove to be a promising strategy for the identification of effective compounds against mycobacterial infections.

## Materials and Methods

### Materials, bacterial strains and growth conditions

Molecular biology methods employed in this study were performed according to the standard protocols by Sambrook and Russell [47]. Plasmid pET16b/thiD and HMP were kind gifts from Prof. T. P. Begley (Department of Chemistry, Texas A&M University). Thiazole-phosphate was synthesized by Jubilant Chemsys Ltd., Noida, India by using the protocol described by Williams et al [48] and Camiener et al [49]. The compounds were obtained from the Drug Synthesis and Chemistry Branch, Developmental Therapeutics Program, Division of Cancer Treatment and Diagnosis, National Cancer Institute, National Institutes of Health. Potassium ferricyanide (K_3_FeCN_6_) and H_2_O_2_ were procured from Thermo Fisher Scientific India Ltd. (Sion, Mumbai, India). Isobutanol was procured from Qualigens fine chemicals (Dr. Annie Besant Road, Mumbai, India) and all other reagents were obtained from Sigma-Aldrich Inc. (St. Louis, MO, USA). Ni-NTA and strep - tactin superflow resin were procured from IBA (Goettingen, Germany). RPMI medium and FCS (fetal calf serum) were obtained from Invitrogen Corporation (Carlsbad, California, USA). *E.coli* BL21 (λDE3) cells were grown in Luria Bertani (LB) broth at 37°C with constant shaking at 200 rpm. *M.smegmatis* mc^2^155 and *M.tuberculosis* H37Rv were grown by using either Difco Middlebrook (MB) 7H9 supplemented with 0.5% glycerol, 0.2% tween−80 at 37°C with constant shaking at 200 rpm or on Difco Middlebrook 7H11 agar. In case of *M.tuberculosis*, the medium was supplemented by Albumin-Dextrose-Catalase (ADC). The Difco Middlebrook media and ADC were obtained from Becton Dickinson and Company (Sparks, MD, USA). Whenever appropriate, antibiotics were added at a concentration of 50 µg/ml ampicillin (Amp) and 25 µg/ml Kanamycin (Kan).

### Cloning and expression of *thiE* gene

*thiE* gene of *M.tuberculosis* (*rv0414c*) was previously cloned in pBEn-SET3a vector at *Bam*HI and *Hind*III sites (unpublished data). The sequence of the gene was derived from EMBL/Genbank. 5′gaattcggatccgctagcgtgcacgaatcccgtctggc3′ containing *Bam*HI and *Nhe*I site was used as the forward primer and 5′gaattcaagcttattatttcgaactgcgggt-ggcccaaagcgctgttcgctgctgtaagcgccgac3′ containing strep tag-stop-*Hind*III site was used as reverse primer for PCR amplification. *thiE* gene was excised from recombinant plasmid pBEn-3a/thiE by restriction digestion with *Nhe*I/*Hind*III restriction enzymes. The resulting *thiE* gene was cloned into pET28a digested with *Nhe*I and *Hind*III and the resulting plasmid pET28a/thiE was used for expression studies. For expression of *thiE*, *E.coli* BL21 (λDE3) cells transformed with pET28a/thiE were grown to mid-logarithmic phase in LB media containing 25 µg/ml of kanamycin and synthesis of MtTPS protein was induced by the addition of 1 mM isoproryl-1-thio-β-D-galactopyranoside (IPTG) followed by harvesting of the cells after incubation at 37°C (3 hours), 25°C (6 hours) or 15°C (16 hours) with a constant shaking at 200 rpm.

### Purification of MtTPS

After induction at 15°C, the *E.coli* cells from a 2 litre LB culture were harvested by centrifugation at 4°C, 6000 g for 10 minutes and resuspended in 25 ml of resuspension buffer (20 mM Tris-HCl, 50 mM NaCl, 1 mM phenylmethylsulfonyl fluoride (PMSF), 2 mM β-mercaptoethanol, pH 8.0) followed by sonication. After sonication, the cell lysate was centrifuged at 12,000 g for 1 hour at 4°C and the supernatant was loaded onto a pre-equilibrated 4 ml strep - tactin superflow column. The column was then washed with 4 column volumes of wash buffer (20 mM Tris-HCl, 50 mM NaCl, pH 8.0) and protein was eluted in 1 ml fractions by using elution buffer (wash buffer +2.5 mM desthiobiotin). After determining the protein concentration by Bradford assay [50], the fractions were analyzed by SDS-PAGE using a 12.5% gel and the fractions containing TPS were pooled and concentrated by using Amicon Ultra protein concentrator (Millipore, Billerica, MA, USA) with a 5 kDa cutoff filter. NaCl and desthiobiotin were removed from the purified sample by passing it through a G-25 column (7 ml) equilibrated with 20 mM Tris-HCl, pH 8.0 and the purified protein was stored at −20°C in aliquots after adding 10% glycerol and 1 mM DTT.

### Purification of *E.coli* ThiD and preparation of HMP-PP

BL21 (λDE3) cells were transformed with plasmid pET16b/thiD (carrying the gene for *E.coli* HMP-kinase or *thiD*) and the transformants were used to inoculate 2 liter LB media with 50 µg/ml ampicillin followed by growth to an A_600nm_ of 0.8–1.0 at 37°C. After 16 hours of induction with 1 mM IPTG at 15°C, the cells were harvested and resuspended in approximately 25 ml of resuspension buffer (20 mM Tris-HCl, 50 mM NaCl, 10 mM imidazole, 1 mM PMSF, 2 mM β-mercaptoethanol, pH 8.0) followed by sonication. After sonication followed by centrifugation for 1 hour at 12,000 g at 4°C, the supernatant was subjected to purification by Ni-NTA affinity chromatography. Briefly, the clarified lysate was mixed with 3 ml Ni-NTA resin, on a rocker for 1 hour at 4°C, unbound proteins removed by centrifugation at 2000 g for 2 minutes and the resin was washed twice with the resuspension buffer. For higher stringency, washings were repeated twice with resuspension buffer containing 20 mM imidazole followed by one more round of washing with resuspension buffer containing 50 mM imidazole. The protein was eluted by using resuspension buffer containing 250 mM imidazole in 1.5 ml fractions followed by protein estimation and dialysis of the pooled fractions against 20 mM Tris-HCl, pH 8.0. The purified protein was stored at −80°C after the addition of 10% glycerol and 1 mM DTT.

The HMP-PP was prepared as described earlier [51]. Briefly, a 0.08 mM stock solution of HMP-PP was prepared by adding 640 µg of HMP-kinase (ThiD) to a 500 µl solution containing 0.1 mM HMP and 2.5 mM ATP in reaction buffer (6 mM MgCl_2_ +100 mM Triethanolamine-HCl, pH 8.0) followed by incubation at 25°C for 30 minutes. ThiD converts HMP to HMP-PP with concomitant production of ADP. The latter can be monitored by its subsequent utilization in an NADH dependent Pyruvate Kinase (PK) and Lactate Dehydrogenase (LDH) coupled reaction [52]. For every 2 molecules of ADP formed, 1 molecule of HMP-PP is generated in the HMP-kinase reaction, which was used in the TPS reaction without further purification as ADP, ATP and HMP- Kinase are known not to interfere in the assay.

### Thiamin phosphate synthase assay

The activity of thiamin phosphate synthase was measured by using the thiochrome assay as described earlier [51], [53]. Briefly, the reaction mixture (500 µl) contained 50 mM Tris-HCl (pH 7.5), 6 mM MgSO_4_, 25 µg MtTPS, 100 µM Thz-P and 100 µM HMP-PP. Reaction was carried out at 37°C for 2 hours. Following this, aliquots of 60 µl were removed, reaction was terminated with 60 µl of 10% TCA followed by centrifugation at 12000 g for 2 minutes to remove the proteins and to 100 µl of each resulting clarified solution, 200 µl of 4 M potassium acetate was added. Potato acid phosphatase (type –II) was then added (100 µl of 2 mg/ml solution) and the mixture was incubated for 1 hr at 37°C. The resulting thiamin (THI) was oxidized to thiochrome by the addition of 100 µl of 3.8 mM K_3_FeCN_6_ in 7 M NaOH, immediately followed by thorough mixing. The reaction was quenched after 30 seconds by the addition of 100 µl of 0.06% H_2_O_2_ in 5.5 M KH_2_PO_4_, diluted with 400 µl of water, and then extracted with 800 µl of isobutanol and centrifuged at 12000 rpm for 10 minutes. After phase separation, 800 µl of the organic phase was transferred to a flourescence cuvette and the thiochrome fluorescence was recorded on fluorimeter (Cary Eclipse Flourescence spectrophotometer, Varian Inc., Santa Clara, CA, USA) with an excitation wavelength of 389 nm and emission wavelength of 434 nm.

### Homology modeling of MtTPS

Amino acid sequence similarity search was performed by using the NCI BLAST server [13] with PDB structure database as the search set. Based on the BLAST results, the first few potential hits were aligned with the MtTPS sequence by using the program ClustalW [16]. The resulting alignments were then submitted for automatic model building of MtTPS by using SWISS-MODEL server [17], [18]. Comparative homology modeling was also attempted by using online software ESyPred3D [19].

### Virtual screening

The NCI diversity set II containing 1541 compounds was selected for virtual screening and docking was performed by using Autodock4 [29] and DOCK6 [30] and re-ranking of the docked ligands was carried out by software X-Score [31]. The three-dimensional co-ordinates for the ligands already processed in the pdbqt format required for autodock were obtained from the autodock database website [54]. The substrate binding cavity or the HMP-PP binding site was targeted for docking. The docking parameters for Autodock4 included the genetic algorithm, 1750000 energy evaluations and 20 runs. Each docking took an average of 10 minutes per processor per ligand. 194 ligands resulted in higher binding energies than the positive control (CF_3_HMP-PP) and the pdbqt files of these docked ligands were extracted from the log files (dlg), which were subsequently converted to pdb format. All the ligands in the pdb format were then written to a single multi-mol2 file using the software chimera [55], which was used for re-ranking by X-Score [31].

The ligands in the pdbqt format obtained from autodock database website [54] were processed in order to use them with software DOCK6 [30]. The 1541 pdbqt files were converted to pdb format and then into a single multi-mol2 file by using the software chimera [55], to which charges and hydrogen atoms were added with Accelrys Discovery Studio 2.5 (Accelrys Software Inc., San Diego 92121, CA, USA) and the final mol2 file was used for virtual screening with DOCK6 [30]. Re-ranking of the results obtained from both the docking softwares was carried out by using the X-Score [31] software using default parameters with the ligand pre-screening option enabled to screen the molecules based on the Lipinsky rules (molecular weight, logP, and number of H-bond donor and acceptor atoms). Only the molecules that complied with the rules or had the drug-like properties were considered in the scoring. Top 40 compounds were requested from NCI/DTP based on the comparison of Autodock4 results, DOCK6 results and X-Score applied to Autodock4 and DOCK6 results and 39 compounds were finally obtained for biochemical screening.

### Inhibitory activity of compounds and determination of IC_50_ values

All the compounds were dissolved in DMSO at a concentration of 1.6 mg/ml. 25 µg of purified MtTPS and 30 µl of respective compounds (final conc. – 100 µg/ml) in a total volume of 105 µl by using reaction buffer were incubated at 37°C for 10 minutes before proceeding with the MtTPS assay as described above. In the control samples, the compounds were replaced with the same volume of DMSO. Compounds displaying >20% inhibition of the enzyme activity were screened at multiple concentrations (5–300 µg/ml) and the activity was measured by using 5 µM of HMP-PP. IC_50_ values (concentration of the compound resulting in 50% inhibition of the enzymatic activity) were determined from the dose-response curves.

### Determination of Minimum Inhibitory Concentration

*M.smegmatis* mc^2^155 or *M.tuberculosis* H37Rv was grown in MB 7H9 - tween media (ADC was added to the media in case of *M.tuberculosis*) till early-logarithmic phase (A_600nm_ of 0.8) and the cells were subsequently diluted to an A_600nm_ of 0.02 (∼2×10^6^ cfu/ml) in respective media. 1 ml aliquots of this culture were incubated with varying concentrations of the compounds along with the controls (containing appropriate concentrations of DMSO) for 24 hours (*M.smegmatis*) or 7 days (*M.tuberculosis*) at 37°C with constant shaking at 200 rpm. The cultures were serially diluted with MB 7H9 media and CFU was determined by plating on MB 7H11 agar plates after incubation at 37°C for 3–4 days (*M.smegmatis*) or 3–4 weeks (*M.tuberculosis*). MIC_99_ value is the concentration of the compound which resulted in 99% inhibition of the growth.

### Cytotoxicity assay

The cytotoxicity of compound 9 was assessed by employing THP-1 (Human acute monocytic leukemia cell line), HeLa (Human cervical cancer cell line), HepG2 (Human liver hepatocellular carcinoma cell line) and HuH (Human hepatocellular carcinoma cell line) cell lines. THP-1 cells were purchased from the National cell repository, National Centre for Cell Science, Pune, India whereas all other cell lines were from American Type Culture Collection (ATCC), Manassas, USA). Cells were seeded at a density of 5×10^4^ cells per well in 1 ml RPMI (for THP-1) or DMEM (for other cell lines) media containing 10% FCS and antibiotic-antimycotic mix in a 24 well plate. The THP-1 cells were activated by the addition of 30 nM Phorbol 12-myristate 13-acetate for 16 hours at 37°C in 5% CO_2_. Cells were allowed to adhere overnight and fresh medium containing various concentrations of compound 9 was added along with the DMSO control. Cells were incubated for 48 hours followed by counting for the viable number of cells by using trypan blue vital dye.

## Supporting Information

Figure S1 **(a) Analysis of expression and localization of MtTPS by SDS - PAGE using a 12.5% gel.** Lane1- whole cell lysate of un-induced sample, Lane 2- insoluble fraction of un-induced sample, Lane 3- soluble fraction of un-induced sample, Lane 4 - molecular weight markers, Lane 5 – whole cell lysate of induced sample, Lane 6 – insoluble fraction of induced sample, Lane 7- soluble fraction of induced sample. (b) Analysis of the purified MtTPS. Lane 1- lysate, Lane 2- unbound proteins, Lane 3- wash, Lane 4- molecular weight markers, Lane 5-10- eluted fractions. (The molecular weight markers comprise of proteins with molecular mass of 97 kDa, 66 kDa, 43 kDa, 29 kDa, 20 kDa and 14 kDa).
(TIF)

Figure S2 **Analysis of the purity of** ***E.coli*** **HMP-kinase (ThiD) by SDS-Polyacrylamide gel electrophoresis using 12.5% gel.** Lane 1- lysate, Lane 2- unbound proteins, Lane 3 – wash with 20 mM imidazole buffer, Lane 4 – wash with 50 mM imidazole buffer, Lane 5- molecular weight markers, Lane 6 -20 – eluted fractions. (The molecular weight markers comprise of proteins of with molecular mass of 97 kDa, 66 kDa, 43 kDa, 29 kDa, 20 kDa and 14 kDa).
(TIF)

Figure S3 **Study of influence of substrate concentrations [HMP-PP (a) and Thz-P (b)] on the activity of MtTPS.**
(TIF)

Figure S4 **Sequence alignment of MtTPS with PfTPS and BsTPS generated by ClustalW.** Identical, conserved substitutions and semi-conserved substitutions of amino acid residues are represented by asterisk (*), colon (:) and dot (.), respectively.
(TIF)

Figure S5 **Ramachandran plot analysis of MtTPS homology model (TPS_1XI3).** 87.5%, 8.7% and 2.2% of the residues are located in the most favoured, additionally allowed and generously allowed regions, respectively with 1.6% of residues in the disallowed region. The plot was generated by web-based PROCHECK.
(TIF)

Figure S6 **Analysis of the Z-score for MtTPS model by using PROSA.** The observed Z-score (black dot) is well within the range of scores typically reported for X-ray crystal and NMR structures of proteins with a similar size.
(TIF)

Figure S7 **Analysis of the local quality for MtTPS model by using PROSA.** The plot showing the average energy over each 40-residue fragment (thick line) confirms the accuracy of the local model quality with negative energy values all throughout the sequence.
(TIF)

Table S1 **List and ranking of top 39 selected compounds obtained from Autodock4, DOCK6 and X-Score results.** NA- Not available in top 40 hits.
(DOC)

Supporting Information S1 **Chemical structures of the top 39 selected compounds.**
(PDF)

## References

[1] Breslow R (1958). On the mechanism of thiamin action. IV. Evidence from studies on model systems.. J Am Chem Soc.

[2] Makarchikov AF, Lakaye B, Gulyai IE, Czerniecki J, Coumans B (2003). Thiamine triphosphate and thiamine triphosphatase activities: from bacteria to mammals.. Cell Mol Life Sci.

[3] Frank RA, Leeper FJ, Luisi BF (2007). Structure, mechanism and catalytic duality of thiamin – dependent enzymes.. Cell Mol Life Sci.

[4] Bettendorff L, Wins P (2009). Thiamin diphosphate in biological chemistry: new aspects of thiamin metabolism, especially triphosphate derivatives acting other than as cofactors.. FEBS J.

[5] Du Q, Wang H, Xie J (2011). Thiamin (Vitamin B1) biosynthesis and regulation: A rich source of anti-microbial drug targets?. Int J Biol Sci.

[6] Backstorm AD, McMordie RAS, Begley TP (1995). Biosynthesis of Thiamin I : The Function of the thiE gene product.. J Am Chem Soc.

[7] Sassetti CM, Rubin EJ (2003). Gentic requirements for mycobacterial survival during infection.. Proc Natl Acad Sci.

[8] Rodionov DA, Vitreschak AG, Mironov AA, Gelfand MS (2002). Comparative Genomics of Thiamin Biosynthesis in Procaryotes.. J Biol Chem.

[9] Begley TP, Downs DM, Ealick SE, McLafferty FW, Van Loon AP (1999). Thiamin biosynthesis in prokaryotes.. Arch Microbiol.

[10] Lawhorn BG, Gerdes SY, Begley TP (2004). A genetic screen for the identification of Thiamin Metabolic genes.. J Biol Chem.

[11] Kayama Y, Kawasaki T (1973). Purification and Properties of thiaminphosphatepyrophosphorylase of *Escherichia coli*.. Arch Biochem Biophys.

[12] Kawasaki T (1979). Thiamin phosphate pyrophosphorylase.. Methods Enzymol.

[13] Altschul SF, Gish W, Miller W, Myers EW, Lipman DJ (1990). Basic local alignment search tool.. J Mol Biol.

[14] Chui HJ, Reddick JJ, Begley TP, Ealick SE (1999). Crystal structure of Thaimin Phosphate Synthase from *Bacillus subtilis* at 1.25Å Resolution.. Biochemistry.

[15] Peapus DH, Chui HJ, Campobasso N, Reddick JJ, Begley TP (2001). Structural Characterization of the Enzyme-Substrate, Enzyme-Intermediate, and Enzyme-Product Complexes of Thiamin Phosphate Synthase.. Biochemistry.

[16] Larkin MA, Blackshields G, Brown NP, Chenna R, McGettigan PA (2007). Clustal W and Clustal X version 2.0.. Bioinformatics.

[17] Arnold K, Bordoli L, Kopp J, Schwede T (2006). The SWISS-MODEL workspace: a web-based environment for protein structure homology modeling.. Bioinformatics.

[18] Scwede T, Kopp J, Guex N, Peitsch MC (2003). SWISS-MODEL: an automated protein homology-modeling server.. Nucleic Acids Res.

[19] Lambert C, Leonard N, De Bolle X, Depiereux E (2002). ESyPred3D: Prediction of proteins 3D structures.. Bioinformatics.

[20] Guex N, Peitsch MC (1997). SWISS-MODEL and the Swiss-Pdb Viewer: An environment for comparative protein modeling.. Electrophoresis.

[21] Laskowski RA, MacArthur MW, Moss DS, Thornton JM (1993). PROCHECK - a program to check the stereochemical quality of protein structures.. J App Cryst.

[22] Sippl MJ (1993). Recognition of Errors in Three-Dimensional Structures of Proteins.. Proteins.

[23] Wiederstein M, Sippl MJ (2007). ProSA-web: interactive web service for the recognition of errors in three-dimensional structures of proteins.. Nucleic Acids Res.

[24] Colovos C, Yeates TO (1993). Verification of protein structures: patterns of nonbonded atomic interactions.. Protein Sci.

[25] Bowie JU, Luthy R, Eisenberg D (1991). A method to identify protein sequences that fold into a known three-dimensional structure.. Science.

[26] Luthy R, Bowie JU, Eisenberg D (1992). Assessment of protein models with three-dimensional profiles.. Nature.

[27] Wallner B, Elofsson A (2003). Can correct protein models be identified?. Protein Sci.

[28] Case DA, Darden TA, Cheatham TE, Simmerling CL, Wang J (2006). AMBER 9, University of California, San Francisco.

[29] Morris GM, Goodsell DS, Halliday RS, Huey R, Hart WE (1998). Automated docking using a Lamarckian genetic algorithm and an empirical binding free energy function.. J Comput Chem.

[30] Lang PT, Brozell SR, Mukherjee S, Pettersen EF, Meng EC (2009). DOCK 6: Combining techniques to model RNA–small molecule complexes.. RNA.

[31] Wang R, Lai L, Wang S (2002). Further Development and Validation of Empirical Scoring Functions for Structure-Based Binding Affinity Prediction. J Comput.. -Aided Mol Des.

[32] Sassetti CM, Boyd DH, Rubin EJ (2003). Genes required for mycobacterial growth defined by high density mutagenesis.. Mol Microbiol.

[33] Li SL, Redei GP (1969). Thiamine mutants of the crucifer, *Arabidopsis*.. Biochem Genet.

[34] Redei GP (1965). Genetic blocks in the thiamine synthesis of the angiosperm *Arabidopsis*.. Amer Jour Bot.

[35] Lester HE, Gross SR (1959). Efficient method for selection of auxotrophs in *Neurospora*.. Science.

[36] Ridgway CJ, Douglas HC (1958). Unbalanced growth of yeast due to inositol deficiency.. J Bacteriol.

[37] Henry SA, Donahue TF, Culbertson MR (1975). Selection of spontaneous mutants of inositol starvation in yeast.. Molec gen Genet.

[38] Culbertson MR, Henry SA (1975). Inositol – requiring mutants of *Saccharomyces cerevisiae*.. Genetics.

[39] Henry SA (1973). Death resulting from fatty acid starvation in yeast.. J Bacteriol.

[40] Pontecorvo G, Roper JA, Hemmons LM, MacDonald KD, Bufton AWJ (1953). The genetics of *Aspergillus nidulans*.. Advan Genet.

[41] Kuraishi H, Takamura Y, Mizunaga T, Uemura T (1971). Factors influencing death of biotin deficient yeast cells.. J Gen Appl Microbiol.

[42] Shimida S, Kuraishi H, Aida K (1972). Unbalanced growth and death of yeast due to pantothenate deficiency.. J Gen Appl Microbiol.

[43] Vilcheze C, Weinrick B, Wong KW, Chen B, Jacobs WR (2010). NAD^+^ auxotrophy is bacteriocidal for the tubercle bacilli.. Mol Microbiol.

[44] DesJarlais RL, Seibel GL, Kuntz ID, Furth PS, Alvarez JC (1990). Structure based design of nonpeptide inhibitors specific for the human immunodeficiency virus 1 protease.. Proc Natl Acad Sci.

[45] Song H, Wang R, Wang S, Lin J (2005). A low-molecular-weight compound discovered through virtual database screening inhibits Stat3 function in breast cancer cells.. Proc Natl Acad Sci.

[46] Gerhard K (2006). Virtual ligand screening: strategies, perspectives and limitations.. Drug Discov Today.

[47] Sambrook J, Russell DW (2001). Molecular Cloning: A Laboratory Manual..

[48] Williams RR, Waterman RE, Keresztesy JC, Buchman ER (1935). Studies of Crystalline Vitamin B_1_. III. Cleavage of Vitamin with Sulfite.. J Am Chem Soc.

[49] Camiener GW, Brown GM (1960). The biosynthesis of thiamine II. Fractionation of enzyme system and identification of thiazole monophosphate and thiamine monophosphate as intermediates.. J Biol Chem.

[50] Bradford MM (1976). A rapid and sensitive method for the quantitation of microgram quantities of protein utilizing the principle of protein-dye binding.. Anal Biochem.

[51] Reddick JJ, Nicewonger R, Begley TP (2001). Mechanistic studies on thiamin phosphate synthase: Evidence for a dissociative mechanism.. Biochemistry.

[52] Reddick JJ, Kinsland C, Nicewonger R, Christian T, Downs TM (1998). Overexpression, purification and characterization of two pyrimidine kinases involved in the biosynthesis of thiamin: 4-amino-5-hydroxymethyl-2-methylpyrimidinekinase and 4-amino-5-hydroxymethyl-2-methylpyrimidinephosphate kinase.. Tetrahedron.

[53] Zhang Y, Taylor SV, Chiu HJ, Begley TP (1997). Characterization of the *Bacillus subtilis thiC* operon involved in thiamine biosynthesis.. J Bacteriol.

[54] AutoDock website.. http://autodock.scripps.edu/resouces/databases.

[55] Pettersen EF, Goddard TD, Huang CC, Couch GS, Greenblatt DM (2004). UCSF Chimera--a visualization system for exploratory research and analysis.. J Comput Chem.

[56] DeLano WL (2002). DeLano Scientific, Palo Alto.

