# Supplementary figures and images for: Identification of Inhibitors against Mycobacterium tuberculosis Thiamin Phosphate Synthase, an Important Target for the Development of Anti-TB Drugs
Source: PLoS One. 2011 Jul 26;6(7):e22441. doi: 10.1371/journal.pone.0022441 (PMC3144219; doi:10.1371/journal.pone.0022441)

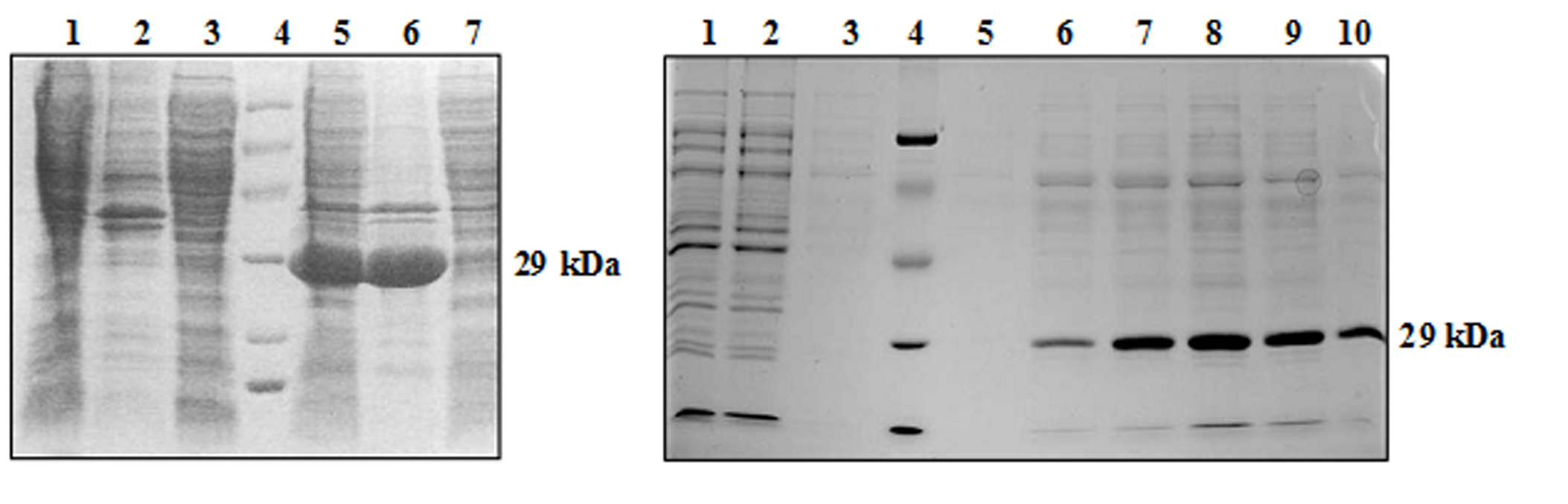

Supplement: Figure S1 — (a) Analysis of expression and localization of MtTPS by SDS - PAGE using a 12.5% gel. Lane1- whole cell lysate of un-induced sample, Lane 2- insoluble fraction of un-induced sample, Lane 3- soluble fraction of un-induced sample, Lane 4 - molecular weight markers, Lane 5 – whole cell lysate of induced sample, Lane 6 – insoluble fraction of induced sample, Lane 7- soluble fraction of induced sample. (b) Analysis of the purified MtTPS. Lane 1- lysate, Lane 2- unbound proteins, Lane 3- wash, Lane 4- molecular weight markers, Lane 5-10- eluted fractions. (The molecular weight markers comprise of proteins with molecular mass of 97 kDa, 66 kDa, 43 kDa, 29 kDa, 20 kDa and 14 kDa). (TIF) [file pone.0022441.s001.tif]

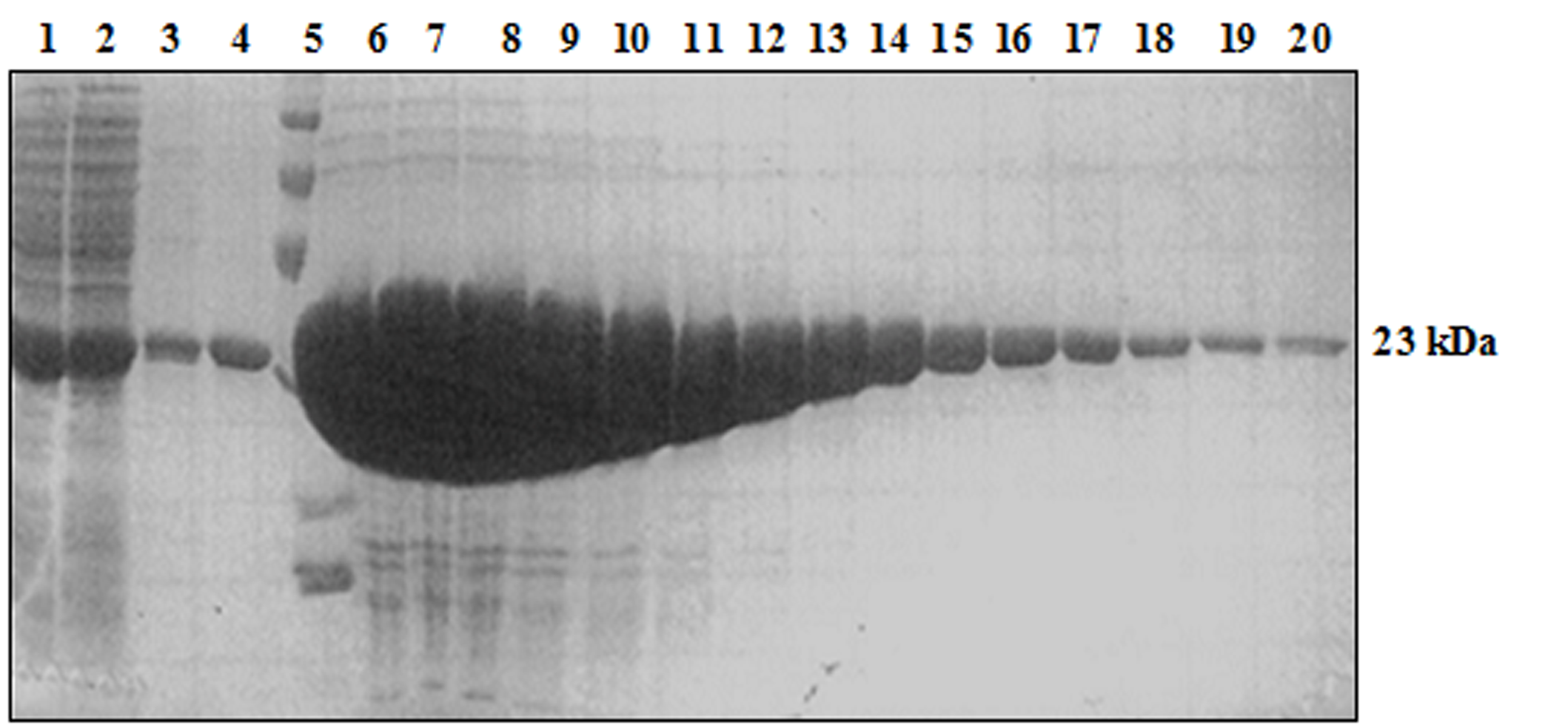

Supplement: Figure S2 — Analysis of the purity of E.coli HMP-kinase (ThiD) by SDS-Polyacrylamide gel electrophoresis using 12.5% gel. Lane 1- lysate, Lane 2- unbound proteins, Lane 3 – wash with 20 mM imidazole buffer, Lane 4 – wash with 50 mM imidazole buffer, Lane 5- molecular weight markers, Lane 6 -20 – eluted fractions. (The molecular weight markers comprise of proteins of with molecular mass of 97 kDa, 66 kDa, 43 kDa, 29 kDa, 20 kDa and 14 kDa). (TIF) [file pone.0022441.s002.tif]

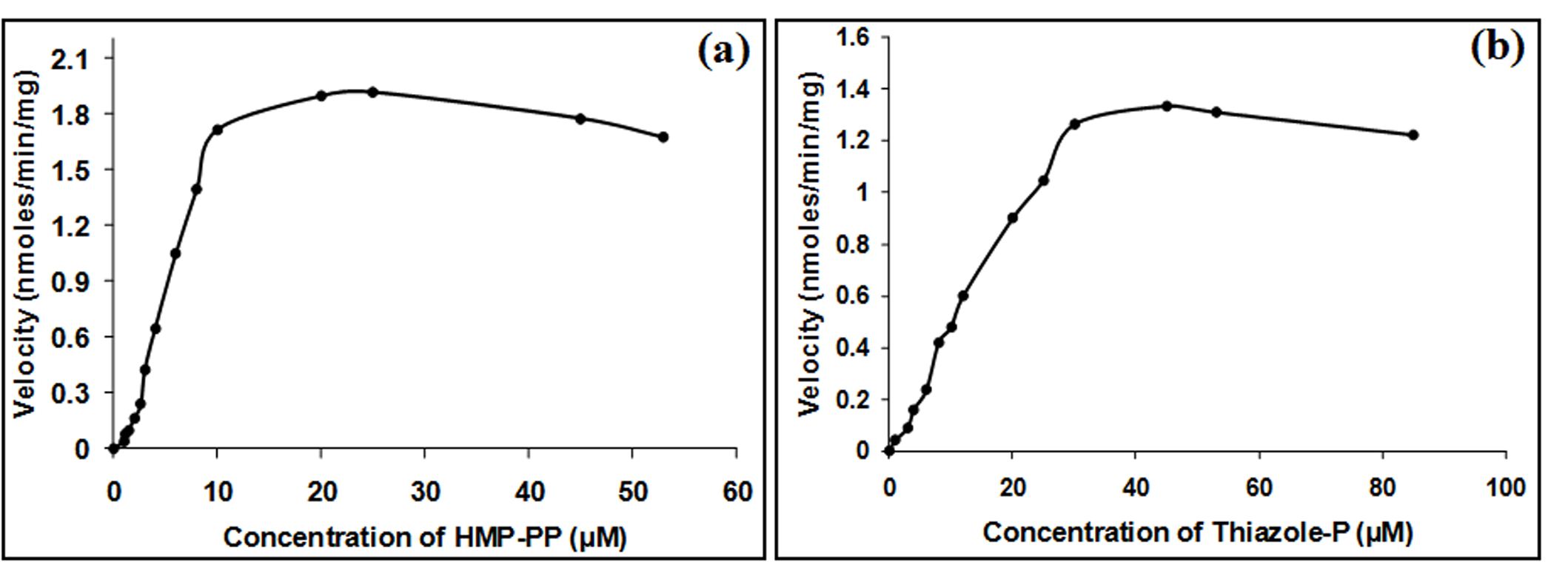

Supplement: Figure S3 — Study of influence of substrate concentrations [HMP-PP (a) and Thz-P (b)] on the activity of MtTPS. (TIF) [file pone.0022441.s003.tif]

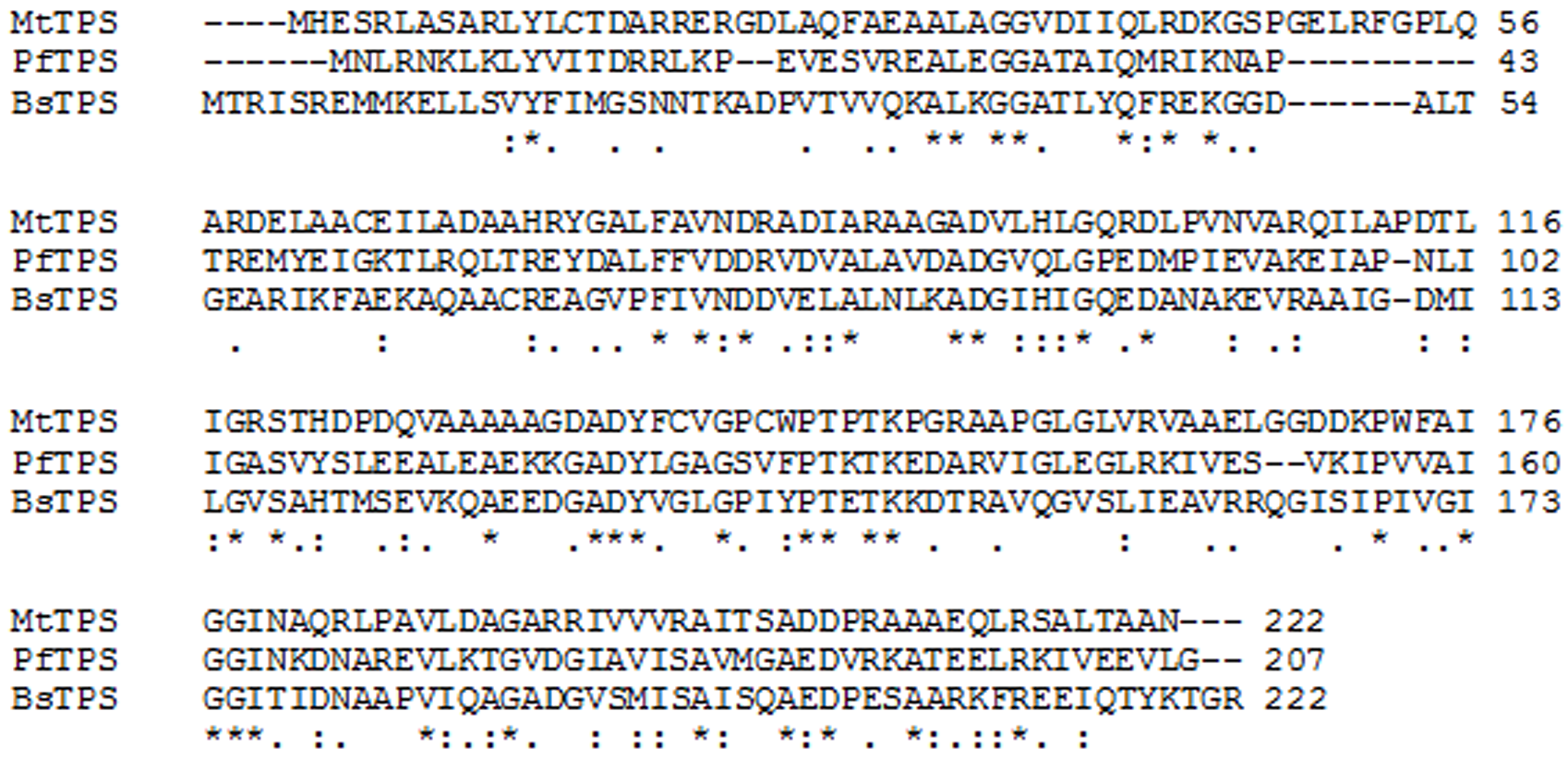

Supplement: Figure S4 — Sequence alignment of MtTPS with PfTPS and BsTPS generated by ClustalW. Identical, conserved substitutions and semi-conserved substitutions of amino acid residues are represented by asterisk (*), colon (:) and dot (.), respectively. (TIF) [file pone.0022441.s004.tif]

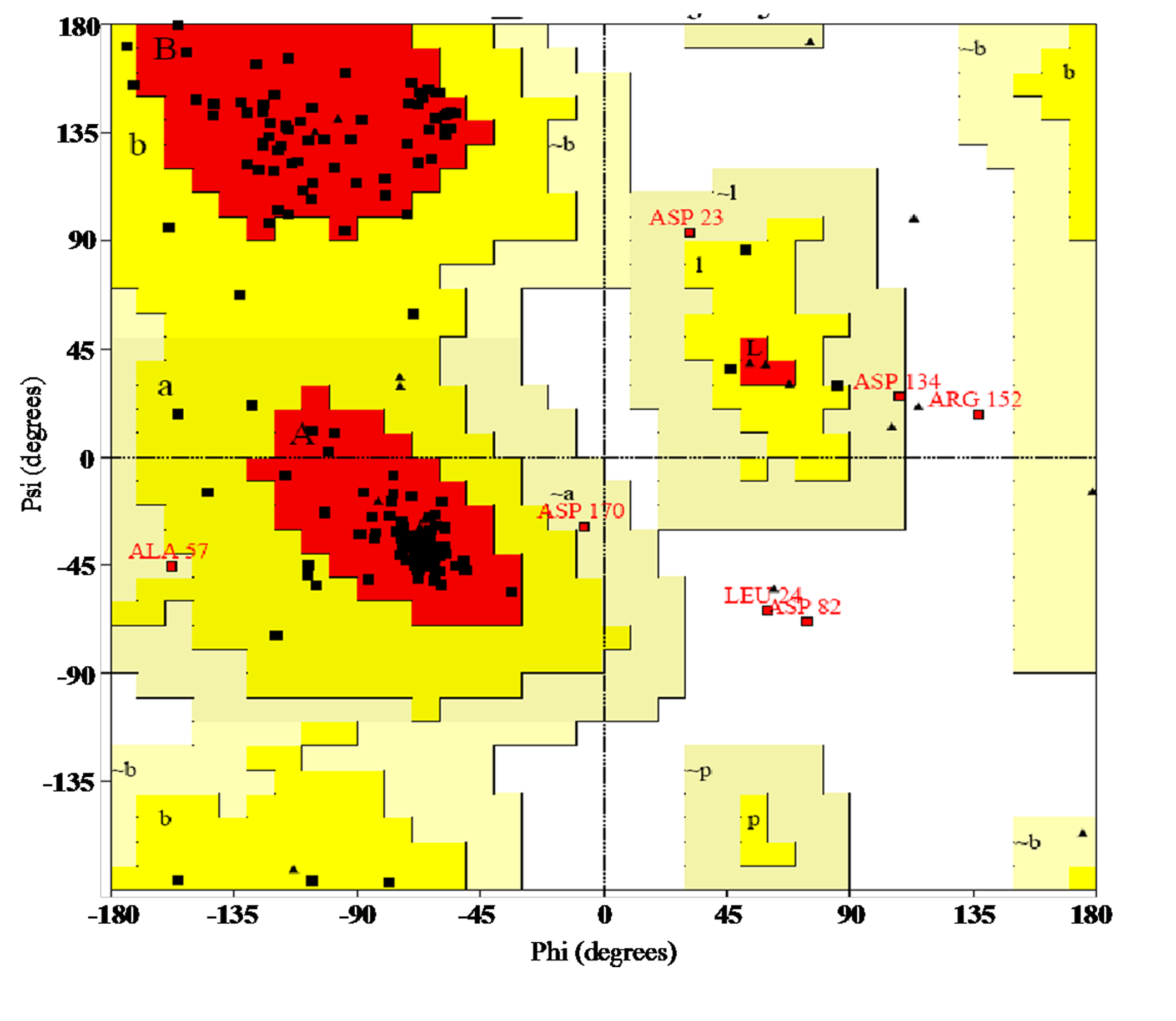

Supplement: Figure S5 — Ramachandran plot analysis of MtTPS homology model (TPS_1XI3). 87.5%, 8.7% and 2.2% of the residues are located in the most favoured, additionally allowed and generously allowed regions, respectively with 1.6% of residues in the disallowed region. The plot was generated by web-based PROCHECK. (TIF) [file pone.0022441.s005.tif]

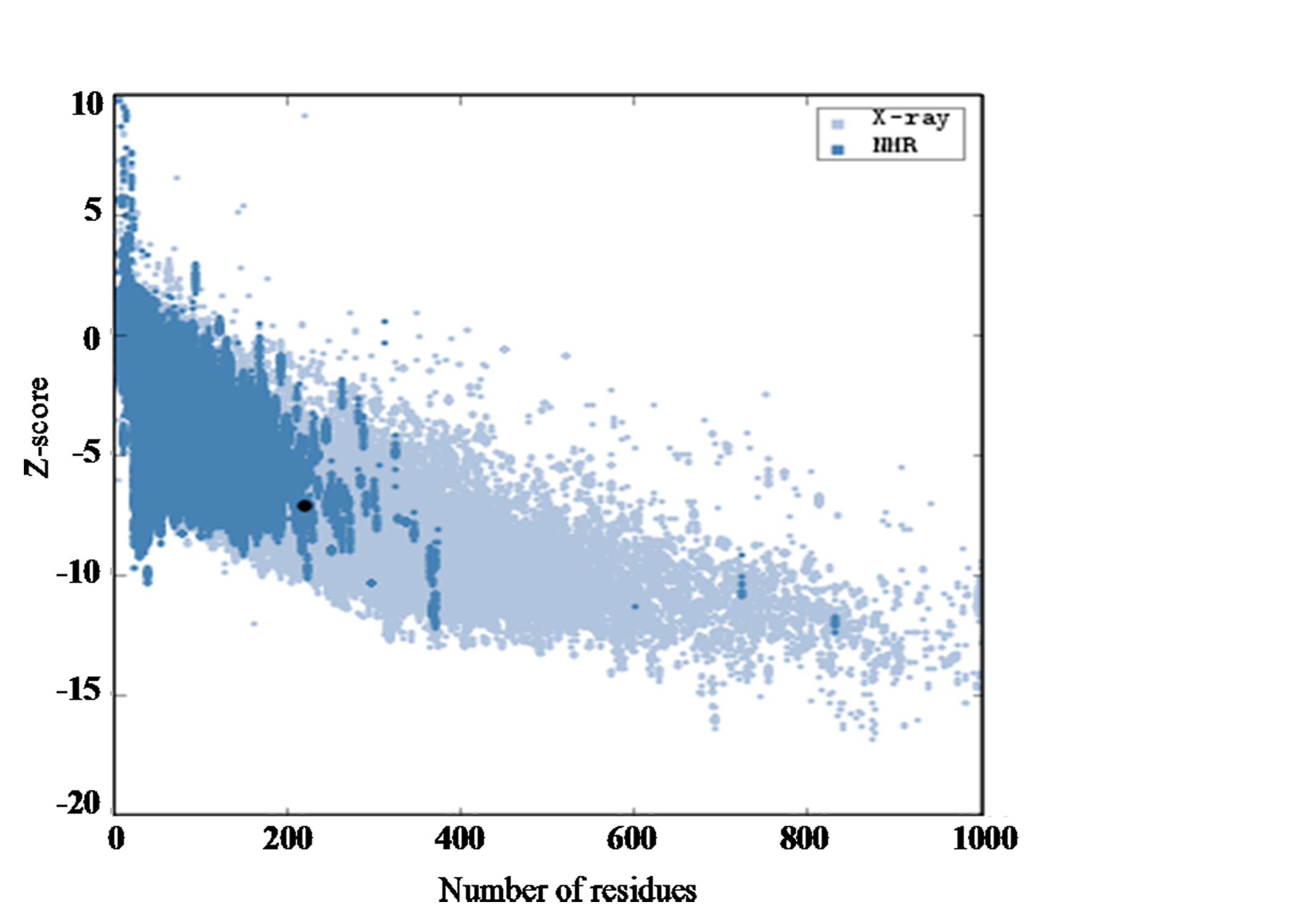

Supplement: Figure S6 — Analysis of the Z-score for MtTPS model by using PROSA. The observed Z-score (black dot) is well within the range of scores typically reported for X-ray crystal and NMR structures of proteins with a similar size. (TIF) [file pone.0022441.s006.tif]

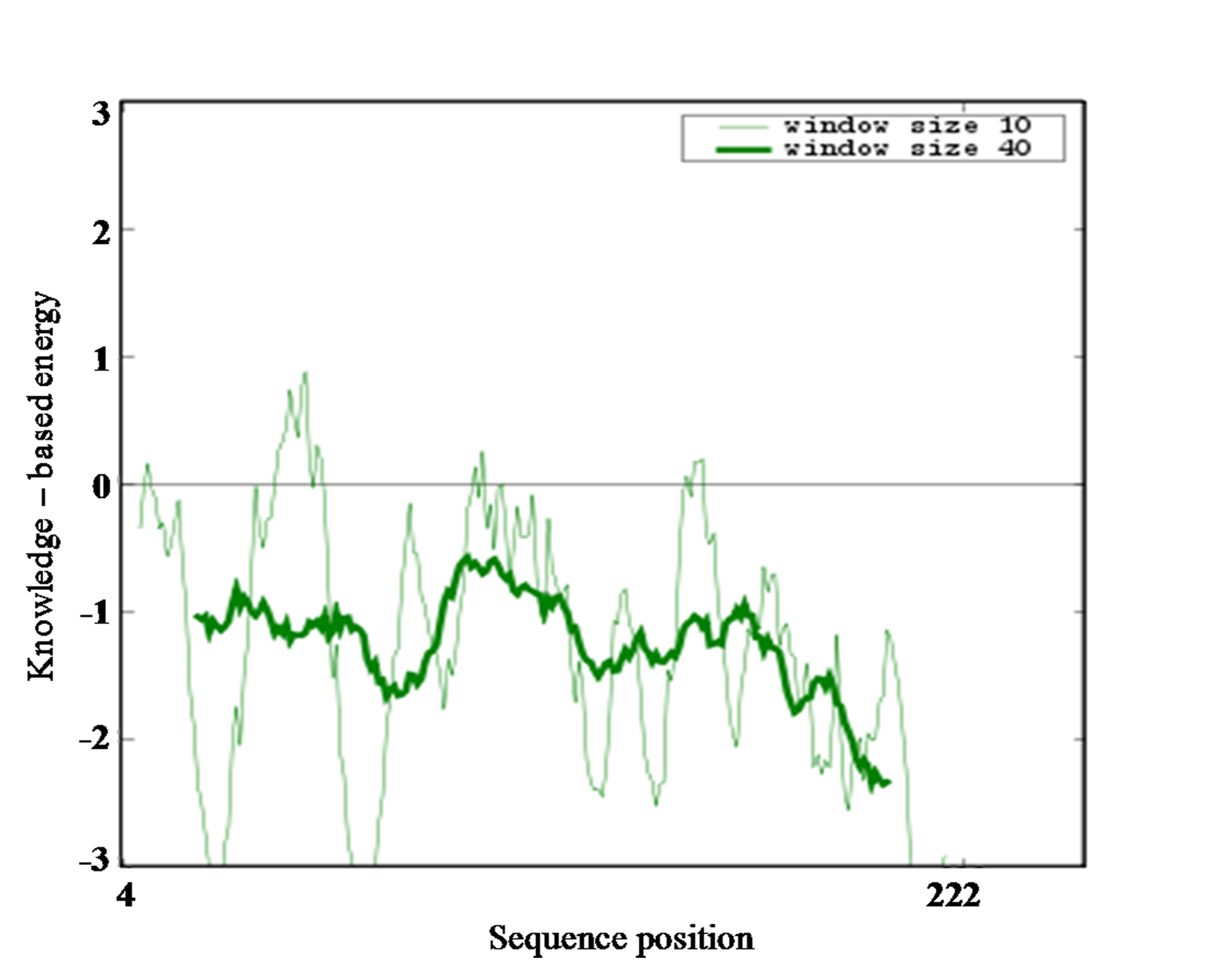

Supplement: Figure S7 — Analysis of the local quality for MtTPS model by using PROSA. The plot showing the average energy over each 40-residue fragment (thick line) confirms the accuracy of the local model quality with negative energy values all throughout the sequence. (TIF) [file pone.0022441.s007.tif]
