# Supplementary material for: Identification of Inhibitors against Mycobacterium tuberculosis Thiamin Phosphate Synthase, an Important Target for the Development of Anti-TB Drugs
Source: PLoS One. 2011 Jul 26;6(7):e22441. doi: 10.1371/journal.pone.0022441 (PMC3144219; doi:10.1371/journal.pone.0022441)
Supplement: Table S1 — List and ranking of top 39 selected compounds obtained from Autodock4, DOCK6 and X-Score results. NA- Not available in top 40 hits. (DOC) [file pone.0022441.s008.doc]

Table S1. List and ranking of top 39 selected compounds obtained from Autodock4, DOCK6 and X-Score results. NA- Not available in top 40 hits.

| **Compd.** | **NSC number** | **Mol. Wt.** | **AutoDock rank** | **DOCK rank** | **XScore (AutoDock ) rank** | **XScore (DOCK) rank** |
| --- | --- | --- | --- | --- | --- | --- |
| 1 | 1614 | 447 | NA | 26 | NA | 28 |
| 2 | 5476 | 334 | NA | NA | 39 | NA |
| 3 | 6821 | 289 | 30 | NA | 7 | NA |
| 4 | 7578 | 343 | 6 | NA | 11 | NA |
| 5 | 13294 | 430 | NA | NA | NA | 2 |
| 6 | 18883 | 349 | NA | NA | 3 | NA |
| 7 | 19061 | 385 | NA | NA | NA | 8 |
| 8 | 26349 | 302 | 29 | NA | NA | NA |
| 9 | 33472 | 314 | 20 | NA | 20 | NA |
| 10 | 37168 | 308 | NA | 16 | 16 | NA |
| 11 | 42199 | 404 | NA | NA | 5 | NA |
| 12 | 50648 | 308 | NA | NA | 15 | NA |
| 13 | 50650 | 363 | NA | 17 | NA | NA |
| 14 | 80997 | 473 | NA | NA | NA | 3 |
| 15 | 85433 | 424 | NA | NA | 6 | NA |
| 16 | 91529 | 516 | NA | 3 | NA | NA |
| 17 | 94914 | 270 | 22 | NA | NA | NA |
| 18 | 96996 | 339 | 3 | NA | 2 | NA |
| 19 | 99634 | 322 | 4 | NA | NA | NA |
| 20 | 109747 | 263 | 18 | NA | 26 | NA |
| 21 | 112541 | 292 | 23 | NA | 10 | NA |
| 22 | 116709 | 353 | 7 | NA | 1 | NA |
| 23 | 116720 | 340 | 2 | 13 | NA | NA |
| 24 | 136513 | 410 | NA | 5 | NA | NA |
| 25 | 156565 | 359 | 5 | NA | 9 | NA |
| 26 | 163910 | 441 | NA | 6 | NA | NA |
| 27 | 191491 | 298 | NA | NA | 23 | NA |
| 28 | 201631 | 436 | NA | 18 | NA | 20 |
| 29 | 211356 | 346 | NA | 8 | NA | NA |
| 30 | 214009 | 362 | NA | 9 | NA | NA |
| 31 | 288027 | 398 | NA | 4 | NA | NA |
| 32 | 321496 | 411 | NA | NA | NA | 6 |
| 33 | 327702 | 353 | 1 | NA | 18 | NA |
| 34 | 328097 | 465 | NA | NA | NA | 7 |
| 35 | 338963 | 422 | NA | NA | NA | 19 |
| 36 | 340852 | 456 | NA | NA | NA | 1 |
| 37 | 359472 | 361 | NA | NA | NA | 38 |
| 38 | 372769 | 290 | NA | NA | 12 | NA |
| 39 | 670283 | 356 | NA | NA | NA | 5 |
