# Supplementary material for: Identification of Inhibitors against Mycobacterium tuberculosis Thiamin Phosphate Synthase, an Important Target for the Development of Anti-TB Drugs
Source: PLoS One. 2011 Jul 26;6(7):e22441. doi: 10.1371/journal.pone.0022441 (PMC3144219; doi:10.1371/journal.pone.0022441)
Supplement: Supporting Information S1 — Chemical structures of the top 39 selected compounds. (PDF) [file pone.0022441.s009.pdf]

Supporting Information S1. Chemical structures of the top 39 selected compounds.

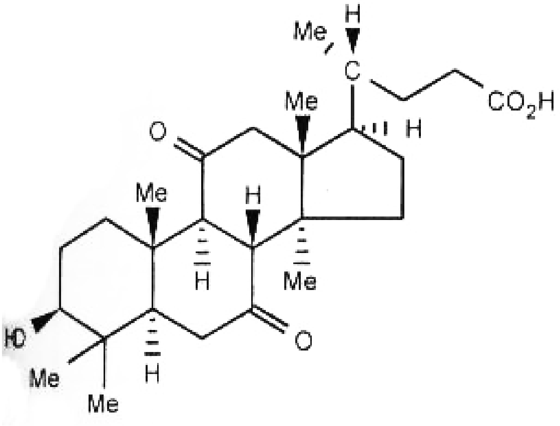

Compound 1, NSC 1614, 3.beta.-hydroxy-7,11-dioxo-4,4,14-trimethyl-5alpha.-cholan-24-oic acid

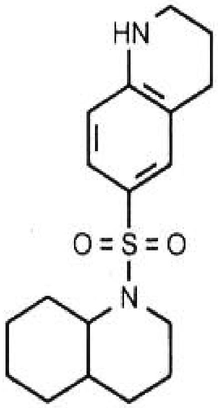

Compound 2, NSC 5476, 6-(octahydroquinolin-1(2H)-ylsulfonly)-1,2,3,4-tetrahydroquinoline

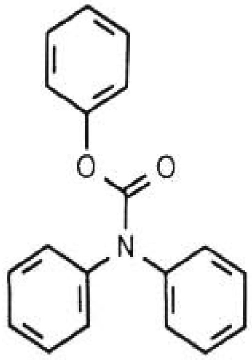

Compound 3, NSC 6821, Carbamic acid, diphenyl-, phenyl ester (8Cl)

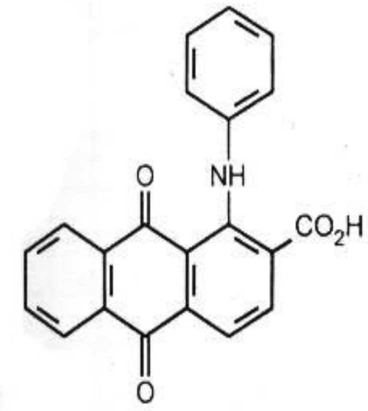

Compound 4, NSC 7578, Red Violet 2RN Acid Anthraquinone

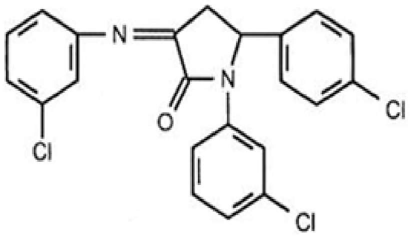

Compound 5, NSC 13294, 1-(3-chlorophenyl)-5-(4-chlorophenyl) -3-[(3-chlorophenyl) imino] pyrrolidin-2-one

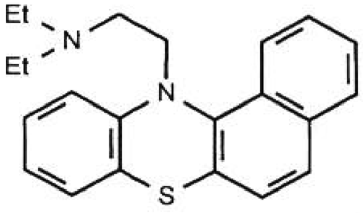

Compound 6, NSC 18883, 2-benzo[a] phenothiazin-12-yl-N,N-diethylethanamine

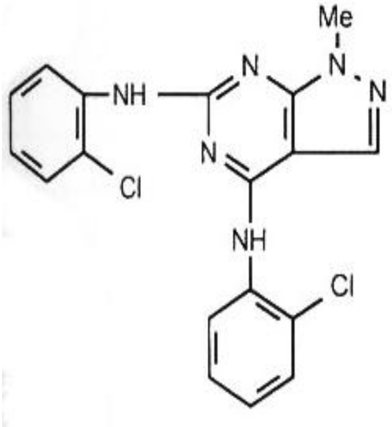

Compound 7, NSC 19061, N,N`-bis(2-chlorophenyl)-1-methyl-1H-pyrazolo[3,4-d]pyrimidine-4,6-diamine

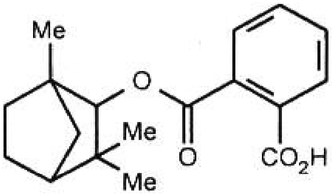

Compound 8, NSC 26349, 2-{[(1,3,3-trimethylbicyclo [2.2.1]hept-2-yl)oxy] carbonyl}benzoic acid

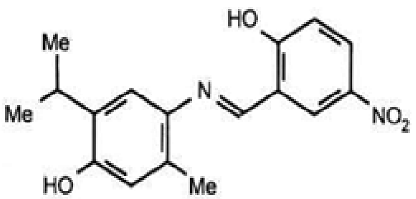

Compound 9, NSC 33472, 4-{[(2-hydroxy-5-nitrophenyl) methylidene] amino}-5-methyl-2-(propan-2-yl) phenol

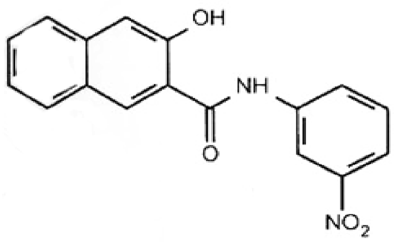

Compound 10, NSC 37168, Naphtazol B

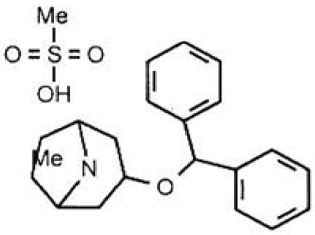

Compound 11, NSC 42199, 3-benzhydryloxy-8-methyl-8-azabicyclo [3.2.1]octane;methanesulfonic acid

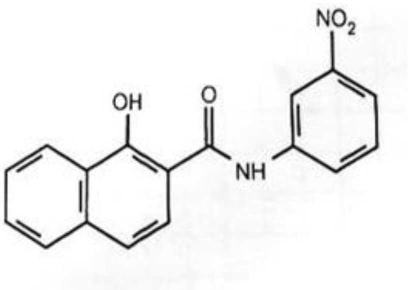

Compound 12, NSC 50648, 1-hydroxy-(3-nitrophenyl) naphthalene-2-carboxamide

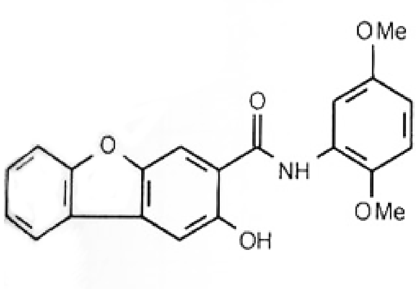

Compound 13, NSC 50650, Naphthanil DB

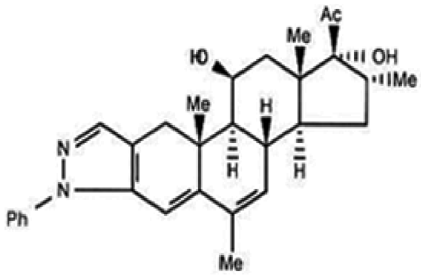

Compound 14, NSC 80997, 2,3-(4`,3`-Pyrazolo)pregna-4,6-dien-20-one, 11.beta.,17-dihydroxy-6,16.alpha.-dimethyl-2`-phenyl-

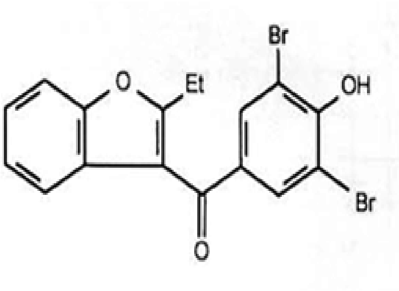

Compound 15, NSC85433, Exurate

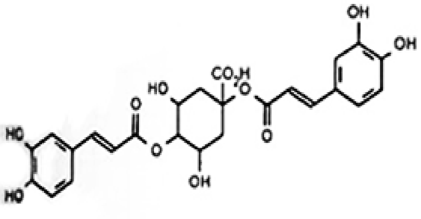

Compound 16, NSC 91529, Cynarin

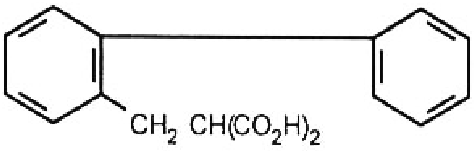

Compound 17, NSC 94914, (biphenyl-2-ylmethyl) propanedioic acid

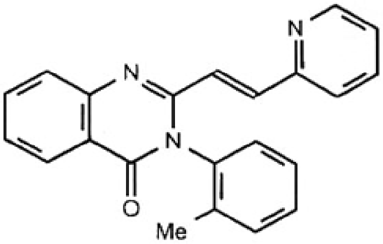

Compound 18, NSC 96996, Piriqualone

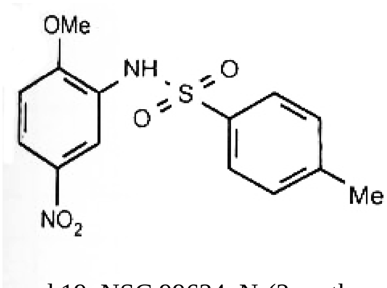

Compound 19, NSC 99634, N-(2-methoxy-5-nitrophenyl)-4-methylbenzenesulfoamide

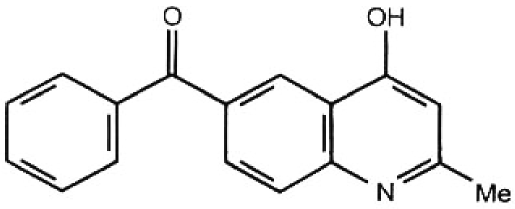

Compound 20, NSC 109747, (4-hydroxy-2-methylquinolin-6-yl)(phenyl) methanone

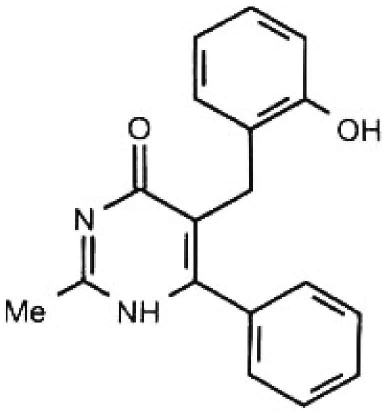

Compound 21, NSC 112541, 5-(2-hydroxybenzyl)-2-methyl-6-phenylpyrimidin-4-one

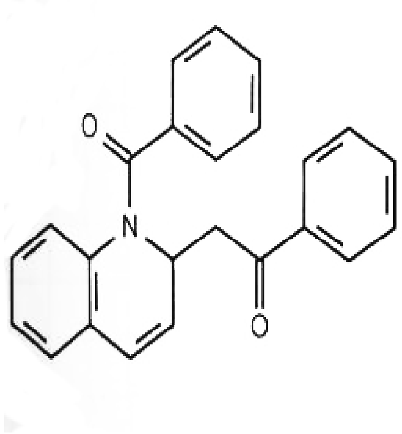

Compound 22, NSC 116709, 1-phenyl-2-[1-(phenylcarbonyl)-1,2-dihydroquinolin-2-yl]ethanone

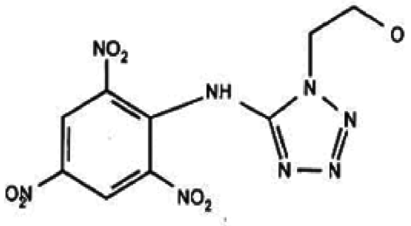

Compound 23, NSC 116720, 2-{5-[2,4,6-trinitrophenyl)amino]-1H-tetrazol-1-yl}ethanol

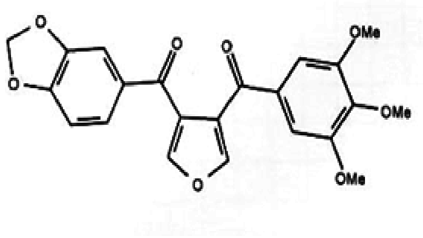

Compound 24, NSC 136513, Furan, 3-piperonyloyl-4-(3,4,5-trimethoxybenzoyl)-(8Cl)

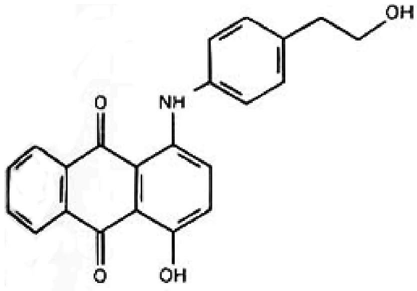

Compound 25, NSC 156565, 1-hydroxy-4-{[4-(2-hydroxyethyl) phenyl] amino} anthracene-9,10-dione

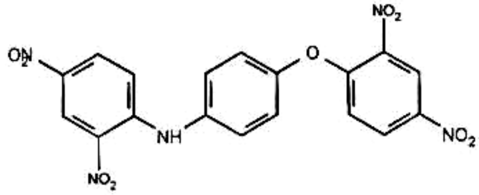

Compound 26, NSC 163910, N-[4-(2,4-dinitrophenoxy) phenyl]-2,4-dinitroaniline

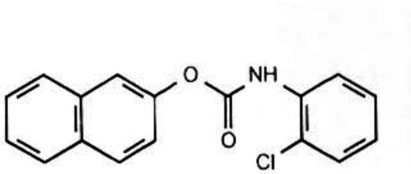

Compound 27, NSC 191491, Naphthalen-2-yl (2-chlorophenyl) carbamate

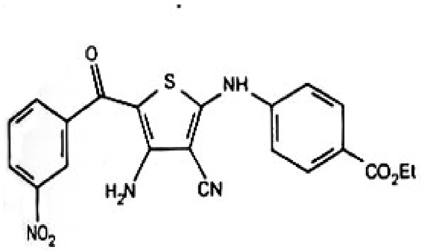

Compound 28, NSC 201631, Ethyl 4-({4-amino-3-cyano-5-[(3-nitrophenyl) carbonyl]thiophen-2-yl} amino) benzoate

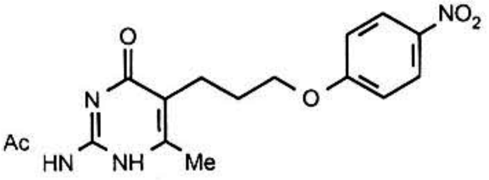

Compound 29, NSC 211356, N-{6-methyl-5-[3-(4-nitrophenoxy)propyl]-4-oxo-1,4-dihydropyrimidin-2-yl}acetamide

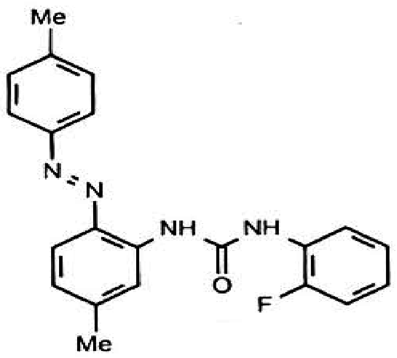

Compound 30, NSC 214009, 1-(2-fluorophenyl)-3-{5-methyl-2-[(4-methylphenyl)diazenyl]phenyl}urea

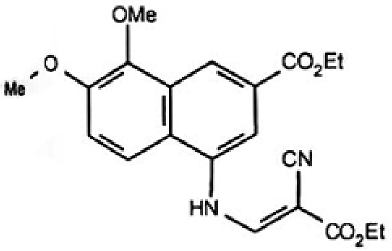

Compound 31, NSC 288027, ethyl 4-[[2-cyano-3-ethoxy-3-oxo-prop-1-enyl] amino]-7,8-dimethoxy-naphthalene-2-carboxylate

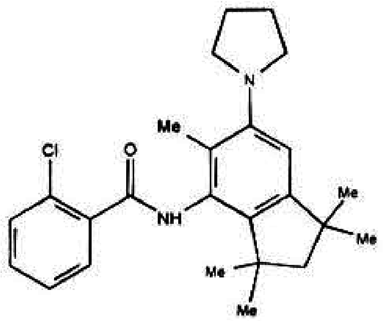

Compound 32, NSC 321496, 2-chloro-N-(1,1,3,3,5-pentamethyl-6-pyrrolidin-1-yl-2H-inden-4-yl)benzamide

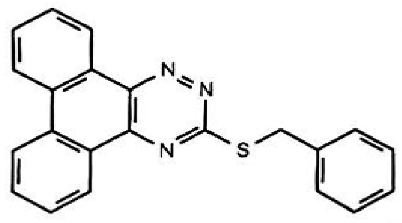

Compound 33, NSC 327702, 3-benzylsulfanyl phenanthro[9,10-e][1,2,4]triazine

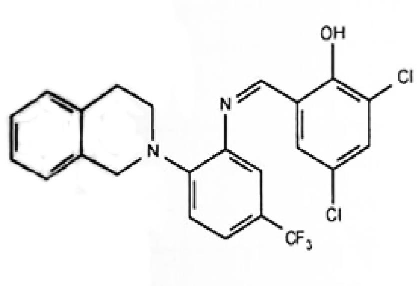

Compound 34, NSC 328097, 2,4-Dichloro-6-[{[2-(3,4-dihydroisoquinolin-2(1H)-yl)-5-(trifluromethyl)phenyl]imino}methyl]phenol

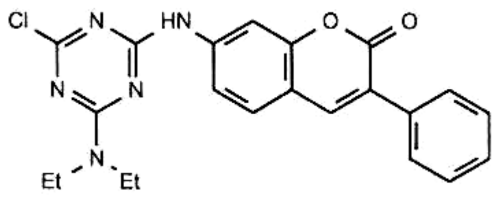

Compound 35, NSC 338963, Coumarin, 7-[[4-chloro-6-(diethylamino)-s-triazin-2-yl]amino]-3-phenyl-

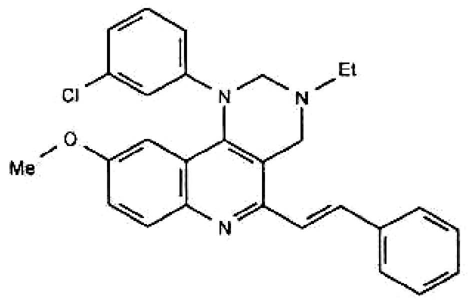

Compound 36, NSC 340852, 1-(3-chorophenyl)-3-ethyl-9-methoxy-5-(2-phenylethenyl)-2,4-dihropyrimido[5,4-c]quinoline

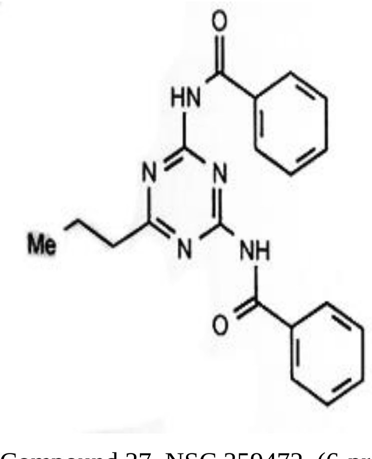

Compound 37, NSC 359472, (6-propyl-1,3,5-triazine-2,4-diyl)dibenzamide

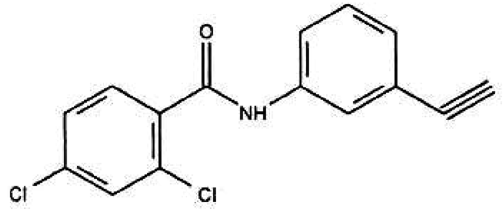

Compound 38, NSC 372769, 2,4-dichloro-(3-ethynylphenyl)benzamide

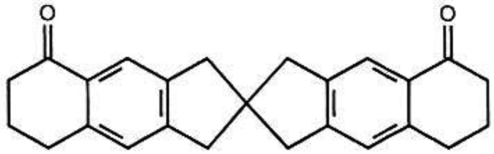

Compound 39, NSC 670283, 2,2`-Spirobi[2H-benz[f]indene]-5,5`-dione, 1,1`,3,3`5,5`6,6`,7,7`,8,8`-dodecahydro-
